# Epstein-Barr Virus Large Tegument Protein BPLF1 Contributes to Innate Immune Evasion through Interference with Toll-Like Receptor Signaling

**DOI:** 10.1371/journal.ppat.1003960

**Published:** 2014-02-20

**Authors:** Michiel van Gent, Steven G. E. Braem, Annemieke de Jong, Nezira Delagic, Janneke G. C. Peeters, Ingrid G. J. Boer, Paul N. Moynagh, Elisabeth Kremmer, Emmanuel J. Wiertz, Huib Ovaa, Bryan D. Griffin, Maaike E. Ressing

**Affiliations:** 1 Department of Medical Microbiology, University Medical Center Utrecht, Utrecht, The Netherlands; 2 Division of Cell Biology, The Netherlands Cancer Institute, Amsterdam, The Netherlands; 3 Department of Biology, National University of Ireland Maynooth, Maynooth, Ireland; 4 Institute of Molecular Immunology, Helmholtz Zentrum München, München, Germany; 5 Department of Molecular Cell Biology, Leiden University Medical Center, Leiden, The Netherlands; University of California Berkeley, United States of America

## Abstract

Viral infection triggers an early host response through activation of pattern recognition receptors, including Toll-like receptors (TLR). TLR signaling cascades induce production of type I interferons and proinflammatory cytokines involved in establishing an anti-viral state as well as in orchestrating ensuing adaptive immunity. To allow infection, replication, and persistence, (herpes)viruses employ ingenious strategies to evade host immunity. The human gamma-herpesvirus Epstein-Barr virus (EBV) is a large, enveloped DNA virus persistently carried by more than 90% of adults worldwide. It is the causative agent of infectious mononucleosis and is associated with several malignant tumors. EBV activates TLRs, including TLR2, TLR3, and TLR9. Interestingly, both the expression of and signaling by TLRs is attenuated during productive EBV infection. Ubiquitination plays an important role in regulating TLR signaling and is controlled by ubiquitin ligases and deubiquitinases (DUBs). The EBV genome encodes three proteins reported to exert *in vitro* deubiquitinase activity. Using active site-directed probes, we show that one of these putative DUBs, the conserved herpesvirus large tegument protein BPLF1, acts as a functional DUB in EBV-producing B cells. The BPLF1 enzyme is expressed during the late phase of lytic EBV infection and is incorporated into viral particles. The N-terminal part of the large BPLF1 protein contains the catalytic site for DUB activity and suppresses TLR-mediated activation of NF-κB at, or downstream of, the TRAF6 signaling intermediate. A catalytically inactive mutant of this EBV protein did not reduce NF-κB activation, indicating that DUB activity is essential for attenuating TLR signal transduction. Our combined results show that EBV employs deubiquitination of signaling intermediates in the TLR cascade as a mechanism to counteract innate anti-viral immunity of infected hosts.

## Author Summary

Epstein-Barr virus (EBV) is a human herpesvirus that persistently infects >90% of adults worldwide. One factor underlying the ability of EBV to establish such widespread and lifelong infections is its capacity to escape elimination by the human immune system. Among the first lines of defense against viral infection is the human Toll-like receptor (TLR) system. These receptors can detect the presence of viruses and initiate an intracellular protein signaling cascade that leads to the expression of immune response genes. The activation status of many proteins in this signaling cascade is regulated by the addition of ubiquitin tags. EBV has previously been reported to encode enzymes, called deubiquitinases (DUBs), which are capable of removing such ubiquitin tags from substrate proteins. In our study, we found that one of these enzymes, BPLF1, functions as an active DUB during EBV production in infected cells before being packaged into newly produced viral particles. Furthermore, our study provides insight into the way in which EBV can subvert the human immune response, as we show that BPLF1 can remove ubiquitin tags from proteins in the TLR signaling cascade. This inhibits TLR signaling and decreases the expression of immune response genes.

## Introduction

Herpesviruses are large enveloped DNA viruses that establish widespread persistent infections. The long coevolution has led to a delicate balance between virus and host. For instance, the human gamma-herpesvirus Epstein-Barr virus (EBV) is carried by over 90% of the adult world population, mostly without overt symptoms [1], even though the virus is also causally involved in infectious mononucleosis and a number of malignancies of lymphoid and epithelial origin [2]. Upon primary infection, EBV establishes a lifelong latent infection in memory B cells, characterized by expression of a limited set of viral gene products. For transmission, viral particles are generated during the productive phase of EBV infection, during which the full repertoire of viral lytic genes is expressed.

To successfully establish infection and replicate, herpesviruses including EBV must withstand elimination by host defense mechanisms. A first line of host defense is posed by the innate immune system. Innate responses are initiated upon recognition of conserved pathogen-associated molecular patterns (PAMPs) by host pattern-recognition receptors (PRRs). Resulting signaling cascades culminate in the production of type I interferons and pro-inflammatory cytokines, whose actions limit viral replication by direct anti-viral effects and through tailoring ensuing adaptive immunity [3]. Among the PRRs contributing to anti-viral immunity are membrane-bound Toll-like receptors (TLRs) and cytosolic RIG-I-like receptors (RLRs).

The importance of TLRs for controlling herpesvirus infection *in vivo* is exemplified by an increased susceptibility to MCMV [4]–[6] or HSV [7], [8] in TLR2, TLR3, TLR7, and/or TLR9 knockout mice as well as in mice lacking the TLR-signaling adaptor MyD88. In humans, genetic studies found an increased incidence of herpesvirus encephalitis in individuals with a defect in the TLR3 pathway, whereas susceptibility to pathogens outside the herpesvirus family was not altered [9]–[13].

TLRs sense PAMPs from a wide variety of pathogens and a number of herpesvirus-derived TLR ligands has now been identified [14]. For EBV, they include virion components that trigger cell-surface displayed TLR2 [15], [16] and virus-derived nucleic acids, such as dsRNA intermediates and genomic DNA, that are sensed by intracellular TLR3, TLR7, and TLR9 [17]–[20].

Upon ligand binding, dimerized TLRs interact with Toll-IL-1 receptor (TIR)-domain containing adaptors [21], [22]. All TLRs except TLR3 recruit adaptor protein MyD88, which leads to phosphorylation of IL-1 receptor-associated kinase (IRAK)-1 and subsequent activation of tumor necrosis factor-associated factor (TRAF)6. To regulate signal transduction and recruit kinase complexes, TRAF6 catalyzes the formation of lysine(K)63-linked polyubiquitin chains on itself and on NF-κB essential modulator (NEMO, or IκB kinase (IKK)γ). Activation of the IKK complex (comprising IKKα, IKKβ, NEMO) then leads to phosphorylation and ubiquitination of the inhibitor of NF-κB, IκBα. This K48-linked ubiquitination targets IκBα for proteasomal degradation, thereby allowing NF-κB to translocate into the nucleus and initiate transcription of genes encoding proinflammatory cytokines. TLR signaling can also lead to the production of type I interferons (IFN). Stimulation of TLR7 and TLR9, expressed by plasmacytoid dendritic cells (pDC) for instance, leads to production of type I IFNs through activation of transcription factor IRF7 (signaled through IRAK1/TRAF6/IKKα). TLR3 or TLR4 ligation results in type I IFNs production following signaling through the adaptor molecule TIR domain-containing adapter inducing IFNβ (TRIF). In addition to this, TRIF-mediated signaling can induce (delayed) activation of NF-κB by triggering TRAF6. These pathways offer several possibilities for interference by (herpes)viruses. Indeed, we have previously reported that mRNA expression levels of several TLRs were reduced in latently and productively EBV-infected B cells [23].

Through post-translational ubiquitin modification, TLR signaling pathways are tightly regulated by cellular ubiquitin ligases and deubiquitinases (DUBs) [24]. The cellular DUBs A20 [25] and CYLD [26] for instance, downmodulate TLR signaling through deubiquitination of TRAF6. Several viruses interfere with the expression of cellular DUBs or encode DUBs themselves [27], [28]. Using probes that identify DUBs by interacting with their catalytic sites, conserved DUB activity was found in the large tegument protein of several herpesviruses [29]–[31]. The EBV homolog of this large tegument protein, BPLF1, consists of 3149 amino acids and its DUB activity is contained in the first N-terminal 204 residues [31]. In the absence of the cloned full-length BPLF1 gene, or BPLF1-specific monoclonal antibodies allowing immunoprecipitation of virally expressed protein, *in vitro* studies so far have used epitope-tagged BPLF1 fragments encoding up to the first 325 most N-terminal amino acids. Besides BPLF1, a bioinformatics screen identified two other EBV proteins with putative DUB activity, BSLF1 and BXLF1 [32].

Since EBV can activate TLR2, TLR3, TLR7, and TLR9, it would be of benefit if EBV were to evade innate immune activation. Here, we examined whether EBV DUB activity was present during productive infection in B cells and whether it could inhibit innate immune signaling.

## Materials and Methods

### Cells

AKBM cells are derived from an EBV^+^ Burkitt's lymphoma (BL) cell line (EBV strain Akata) by stable transfection with the plasmid pHEBO-prBMRF1-ratCD2-GFP yielding inducible rat CD2-GFP expression in productively EBV-infected cells (see below) [33]. AK31 cells are derived from an EBV-negative subclone of the parental Akata BL cell line by stable transfection to achieve constitutive expression of the ratCD2-GFP fusion protein in part of the population. Jijoye cells are EBV^+^ B cells that express LMP1 [34]. AKBM, AK31, and Jijoye cells were maintained in RPMI 1640 medium (Invitrogen) supplemented with 10% FBS (Sigma), 2 mM *L*-glutamine, 100 U/mL penicillin, 100 µg/mL streptomycin, and 0.3 mg/mL hygromycin B (AKBM cells) or 1 mg/mL geneticin (AK31 cells).

Human embryonic kidney 293T cells were maintained in Dulbecco's modified Eagle medium (DMEM; Invitrogen) supplemented with 10% FBS (Sigma) and 2 mM L-glutamine (Invitrogen). 293 cells expressing human TLR2 and CD14 (293-TLR2/CD14) or CD14 alone (293-CD14) were described previously [35] and 293 cells expressing human TLR3 (293-TLR3) were obtained from InvivoGen (San Diego, CA).

### Productive EBV infection and EBV particles

Productive EBV infection was induced in AKBM cells as described [33]. Briefly, B cell surface IgG was cross-linked by incubation with 50 µg/mL goat F(ab′)_2_ fragments to human IgG (Cappel; MP Biomedicals) for the times indicated. Percentages of productively infected cells were determined based on induced expression of the rat CD2-GFP reporter protein. To discriminate (immediate) early and late stages of productive infection, viral DNA replication and late phase gene expression were inhibited by treatment with 300 µg/mL phosphonoacetic acid (PAA, pH 7.4 in 100 mM HEPES, Sigma Aldrich) starting 1 hour prior to anti-IgG treatment. To exclude secondary effects of antibody (Ab) treatment or the presence of PAA, control EBV^−^ AK31 cells were included in parallel.

Directly pelleted EBV (strain B95.8) was purchased from Advanced Biotechnologies Inc. (batch #106-176) and lysates of 5×10^10^ particles/mL were prepared in reducing sample buffer (200 mM Tris-HCl [pH 6.8], 3% SDS, 10% glycerol, 1 mM EDTA, 50 mM DTT, 0.05% bromophenol blue).

### Plasmids

BPLF1 (aa 1–325), BSLF1, or BXLF1 in frame with an N-terminal HA- (YPYDVPDYA) or Flag- (DYKDDDDK) tag were PCR amplified from EBV B95.8 genomic DNA using the primers listed in Table 1. An additional Flag-tagged BPLF1 (aa 1–325) construct was generated by PCR amplification based on EBV strain Akata. Fragments were cloned into Gateway donor vectors and subcloned into the mammalian expression vector pMaxCloning (Lonza). Full-length BPLF1 (FL BPLF1) with an N-terminal Flag-tag and C-terminal HA-tag was engineered in pMaxCloning by joining three overlapping fragments of B95.8 BPLF1 amplified from genomic DNA (see Table 1), using unique restriction sites present inside the BPLF1 open reading frame (EcoRI and ClaI). The resulting sequence was identical to the B95.8 BPLF1 open reading frame except for a deletion spanning nucleotides 1102–1131 (two out of eight PASAA repeats) and a T to A mutation at nucleotide position 1192. BPLF1 (aa 1–325) mutants C61A (TGC to GCC), D86/90R (two times GAC to CGC), or D86/90G (two times GAC to GGC) were generated by site-directed mutagenesis using Pfu Turbo polymerase (Agilent Technologies) and the primers listed in Table 1. Correctness of all constructs was verified by sequencing.

**Table 1:** Primer sequences for cloning EBV putative DUBs.

Gene product	Amino acids	F/R*	Primer sequence (5′-3′)
Flag-FL BPLF1-HA	2–815	F	GGGGTACCCAATTGCCACCATGGATTACAAGGATGACGACGATAAGAGTAACGGCGACTGGGGGCAAAGC
		R	GTGGGGACCTGCAGATGTCCCG
	671–1339	F	GTCCGACTCTGAAGAAGCGGAGAGCG
		R	ATAGTTTAGCGGCCGCTTAGGATGAGCGTTTGGGAGAGCTGATTCTGC
	1189–3149	F	CGGGGCCGCAAAGAACCCCACG
		R	ATAGTTTAGCGGCCGCTTAAGCGTAATCTGGAACATCGTATGGGTACAGATACAAAAACTTGAGTCTCTCGAGG
Flag-BSLF1	2–874	F	GGGCTCGAGACCATGGATTACAAGGATGACGACGATAAGTCCGCCCCCGTCGTCATCAAGG
		R	GGGTCTAGACTAGTTCGGGAGAGTCTCTGAGAAG
Flag-BXLF1	2–607	F	GGGGACAAGTTTGTACAAAAAAGCAGGCTTCCTCGAGACCATGGATTACAAGGATGACGACGATAAGGCTGGATTTCCAGGAAAGGAGGCCGCTGGATTTCCAGGAAAGGAGGCC
		R	GGGGACCACTTTGTACAAGAAAGCTGGGTCTCTAGACTAGTCCCGATTTCCCCTCTC
Flag-BPLF1	2–325	F	GGGGACAAGTTTGTACAAAAAAGCAGGCTTCCTCGAGACCATGGATTACAAGGATGACGACGATAAGAGTAACGGCGACTGGGGGCAAAGC
		R	GGGGACCACTTTGTACAAGAAAGCTGGGTCTCTAGACTAAGGACTATACCTGGCGGCAGGG
HA-BPLF1	2–325	F	GGGGTACCCAATTGCTCGAGCCACCATGTACCCATACGATGTTCCAGATTACGCTAGTAACGGCGACTGGGGGCAAAGC
		R	GGGGACCACTTTGTACAAGAAAGCTGGGTCTCTAGACTAAGGACTATACCTGGCGGCAGGG

* F: forward, R: reverse.

Other plasmids used were: pcDNA3-Flag-MyD88 (M. Muzio, Milan, Italy), IRAK1, Flag-TRAF6 (Tularik), pcDNA3-Flag-IKKα (A. Baldwin, Chapel Hill, USA), IκBα, His-NEMO (IKKγ), Flag-A20, pcDNA3.1-HA-ubiquitin (T. Chiba, University of Tsukuba, Japan), and pUNO-hTLR2 (InvivoGen).

### Transfections

To transiently express individual EBV proteins, 293T cells (1.5×10^6^) were transfected in suspension with a total of 4 µg vector DNA using Lipofectamine 2000 according to manufacturer's instructions (Invitrogen) and seeded in 6-well plates. 16 hours post-transfection, cells were lysed for DUB profiling or immunoblotting (see below).

To visualize general protein ubiquitination, 293T cells (1.5×10^6^) were transfected with a combination of plasmids encoding HA-Ub (1 µg) and BPLF1 variants (2 µg), supplemented with empty vector (1 µg). Cells were seeded in 6-well plates and lysed 40 hours post-transfection in reducing sample buffer (200 mM Tris-HCl [pH 6.8], 3% SDS, 10% glycerol, 1 mM EDTA, 50 mM DTT, 0.05% bromophenol blue).

To study IκBα degradation, 293T cells (1×10^6^) were transfected in suspension using 3 µg DNA and 13 µL PEImax (Brunschwig Chemie). After 40 hours, cells were stimulated with 10 ng/mL MALP-2 and post-nuclear lysates were prepared.

### DUB profiling

Active DUBs were labeled in cell lysates as previously described [36]. In brief, post-nuclear cells were prepared in lysis buffer (50 mM Tris-HCl, 250 mM sucrose, 5 mM MgCl_2_, 1 mM DTT, 0.5% CHAPS, and 0.1% NP40). DUB labeling was performed for 30 min at 37°C in lysis buffer containing 1 mg/mL protein extract, 1 µM 5-carboxytetramethylrhodamine (TMR)-conjugated ubiquitin-based probe, and two equivalents (v/v relative to probe) 50 mM NaOH to neutralize the pH. Reactions were terminated by the addition of reducing sample buffer prior to heating at 90°C for 10 min, after which proteins were resolved by SDS-PAGE (4%–12% gradient gel) and visualized by in-gel fluorescence imaging. Where indicated, gels were further used for immunoblotting.

### Antibodies (Abs)

BPLF1-specific rat monoclonal Abs were prepared by immunizing Lou/C rats with OVA-coupled peptides encompassing BPLF1 amino acids 2–17 (peptide A: SNGDWGQSQRTRGTGP) and amino acids 78–94 (peptide B: PLTSRPELDEVLDEGAR) of B95.8.

To detect other EBV proteins, the following Abs were used: mouse anti-BZLF1, mouse anti-BGLF5 (311H) [37], rabbit polyclonal anti-gp42 (PB1112), and mouse anti-LMP1 (clone CS1-4). Other Abs included: mouse anti-Flag (M2, F3165, Sigma-Aldrich), rat anti-HA (3F10, 11867423001, Roche Diagnostics), mouse anti-His (5H1, HyTest Ltd), mouse anti-β-actin (Clone C4, MAB1501R, Millipore), mouse anti-transferrin receptor (H68.4, 13–6800, Life Technologies), mouse anti-p97 (#612183, BD Transduction laboratories), and rabbit anti-histon H3 (Cell Signaling Tech #9715). Specific Abs against signaling intermediates included rabbit anti-TRAF6 (H274, sc-7221, Santa Cruz, for immunoprecipitation) and mouse anti-TRAF6 (sc-8409, Santa Cruz, for immunoblotting), rabbit anti-NEMO (sc-8330, Santa Cruz), rabbit anti-IκBα (sc-203, Santa Cruz), and rabbit anti-NF-κB (sc-372, Santa Cruz).

For flow cytometry, goat anti-mouse allophycocyanin-conjugated (APC; M101, Leinco Technologies) or fluorescein isothiocyanate-conjugated (FITC; Dako) Abs were used as second step. For immunoblotting, HRP-conjugated secondary Abs used were: anti-rat Ig (light-chain specific, #112-035-175, Jackson), anti-mouse Ig (light-chain specific, #115-035-174, Jackson), and anti-rabbit Ig (4030-05, Southern Biotech). Secondary Abs used for immunofluorescence were anti-Mouse Ig-Cy3 (715-165-151, Jackson), anti-Rabbit Ig-Dylight 488 (711-485-152, Jackson), and anti-Rat Ig-Dylight 488 (712-485-153, Jackson).

### Flow cytometry

For intracellular staining, cells were fixed with PBS/1% formaldehyde and permeabilized using 0.5% saponin (Sigma-Aldrich). Samples were measured on a FACSCantoII or FACSCalibur flow cytometer (BD Biosciences) and analyzed using FACSDiva (BD Biosciences) and FlowJo software (Tree Star).

### Immunoblotting

Immunoblot analysis was performed as described [33]. In brief, post-nuclear cell lysates were prepared as follows: cells were lysed using NP40 lysis buffer (0.5% Igepal-CA630, 50 mM Tris HCl [pH 7.5], 150 mM NaCl, 10 µM Leupeptin, and 1 mM 4-(2-aminoethyl)benzenesulfonyl fluoride), post-nuclear lysates were clarified by centrifugation, and proteins in the lysates were denatured with reducing sample buffer. Where indicated, nuclear fractions were prepared by resuspending the nuclear pellet obtained after centrifugation in NP40 lysis buffer through sonification. Solubilized proteins were resolved by SDS-PAGE and transferred to polyvinylidene difluoride (PVDF) membranes (Bio-Rad). Membranes were incubated with specific primary Abs followed by HRP-conjugated secondary Abs and reactive protein bands were visualized using ECL Plus detection kit (GE Healthcare) and ImageQuant LAS 4000 imager (GE Healthcare Life Sciences). Densitometric quantification of staining intensity was performed using Quantity One software (Bio-Rad).

### Luciferase reporter assays and IL-8 ELISA

Transfections were conducted in suspension using Lipofectamine 2000 (Invitrogen) according to the manufacturer's protocol in 96-well flat-bottom plates. Briefly, 293(T) cells (5×10^4^) were transfected with a total of 200 ng DNA containing 75 ng pGL3-firefly luciferase controlled by either five NF-κB binding sites (NF-κB-luc), part of the human E-selectin promoter (pELAM-luc), or the human IFN-β promoter together with 4 ng of HSV thymidine kinase driven *Renilla* luciferase for normalization (pGL3-TK vector), and indicated amounts of plasmids encoding EBV genes or controls. Where indicated, 293T cells were cotransfected with 5 ng pUNO-TLR2. TLR signaling was induced either by cotransfecting activator proteins MyD88 (15 ng), IRAK1 (8 ng), TRAF6 (40 ng), or IKKα (75 ng) or by treatment for 7 hours with 10 ng/mL TLR2 agonist MALP-2 (sc-221869, Santa Cruz), 10 µg/mL TLR3 agonist poly(I∶C) (HMW, InvivoGen), or 6.5×10^6^ EBV particles/mL (purified virus, batch 4H0012-PV, Advanced Biotechnologies Inc) starting at 16 hours post-transfection. Cells were lysed in Passive Lysis Buffer and Firefly and *Renilla* luciferase activities were measured using the Dual-Luciferase Reporter Assay System according to manufacturer's instructions (Promega). Data were normalized for *Renilla* luciferase activity and presented as percentage firefly luciferase activity relative to the stimulated transfected control sample, mean ± S.D.

To study effects on cellular IL-8 production, 293-TLR2/CD14 cells were transfected in 96-well flat-bottom plates with 200 ng EBV or control plasmids and were stimulated with 10 ng/mL MALP-2 or 6.5×10^6^ EBV particles/mL (batch 4H0012-PV) at 24 hours post-transfection in 100 µL DMEM (transfection efficiencies around 60%). Following 8 hours of stimulation, levels of IL-8 secreted in the culture supernatants were measured by ELISA using PeliKine Compact human IL-8 ELISA kit (Sanquin Reagents) according to manufacturer's instructions.

### Cellular deubiquitination assay

293T cells were transfected with HA-ubiquitin together with either Flag-TRAF6 or His-NEMO and empty vector, Flag-BPLF1, Flag-BPLF1_C61A_, or Flag-A20. Post-nuclear extracts of transfected cells were prepared in lysis buffer (50 mM Tris-HCl [pH 7.5], 150 mM NaCl, 0.5% Igepal [w/v], 1 mM dihiothreitol [DTT], 1 mM phenylmethylsulfonyl fluoride (PMSF), 25 µg/mL Leupeptin, 25 µg/mL aprotinin, 1 mM benzamidine, 10 µg/mL trypsin inhibitor), in case of TRAF6 supplemented with 50 mM NaF and 1 mM Na_3_VO_4_. Samples containing TRAF6 were denatured by addition of SDS to a final concentration of 1% and heating at 95°C for 5 minutes, after which SDS was diluted by adding additional lysis buffer. TRAF6 was precipitated by incubating with anti-TRAF6 Ab overnight at 4°C with constant agitation and for an additional 5 hours with protein A-sepharose beads. His-NEMO was precipitated by incubation with nickel-NTA agarose beads (Qiagen) overnight at 4°C. After washing, precipitated proteins were eluted in reducing sample buffer and analyzed by immunoblotting.

### *In vitro* deubiquitination of signaling intermediates

293T cells were cotransfected with HA-ubiquitin and either Flag-TRAF6, His-NEMO, or IκBα. 24 hours post transfection, IκBα-transfected cells were additionally treated with 50 µM MG132 and 10 ng/mL TNFα for 2 hours. Post-nuclear cells lysates were prepared using NP40 lysis buffer with protease inhibitor cocktail (Roche) and 20 mM NEM. To precipitate (ubiquitinated) TRAF6, NEMO, or IκBα, cell lysates were incubated overnight at 4°C with constant agitation with anti-Flag M2 Abs and protein G sepharose beads, nickel NTA beads (Qiagen), or anti-IκBα Abs and protein A sepharose beads, respectively. Flag-TRAF6(-Ub_n_) was released from the beads using 1 µg/mL Flag peptide (Sigma) and His-NEMO(-Ub_n_) was eluted using 250 mM imidazole. The thus obtained ubiquitinated signaling proteins were incubated for 4 hrs at 37°C in DUB assay buffer (50 mM HEPES-NaOH pH 8.0, 10% glycerol, 3 mM DTT) with Flag-tagged BPLF1, BPLF1_C61A_, or A20 DUB proteins that were purified from separately transfected 293T cells using anti-Flag Abs and protein G sepharose beads. Samples were analyzed by immunoblot analysis.

### Immunofluorescence

293T cells (30.000) were transfected with plasmids encoding BPLF1 and signaling intermediates (125 ng each) using Lipofectamine 2000 and cultured overnight in Lab-Tek II chamber slide system (Thermo Scientific). Cells were fixed using 3% paraformaldehyde in PBS for 30 minutes at 37°C and permeabilized by incubation with PBS/1% Triton X-100/0.1% sodium citrate for 5 minutes at room temperature. After blocking for 60 minutes with PBS/5% BSA, samples were incubated with primary Abs in PBS/1% BSA/0.1% Triton X-100 for 60 minutes, washed, and incubated for 60 minutes with fluorescently labeled secondary Abs together with DNA stain TO-PRO-3 (Life Technologies). Slides were embedded in Mowiol (Brunschwig Chemie) and analyzed using a confocal microscope (Leica SP5) and Leica Application Suite Advance Fluorescence software.

## Results

### The large tegument protein BPLF1 acts as an active deubiquitinase during productive EBV infection

**Figure 1:**
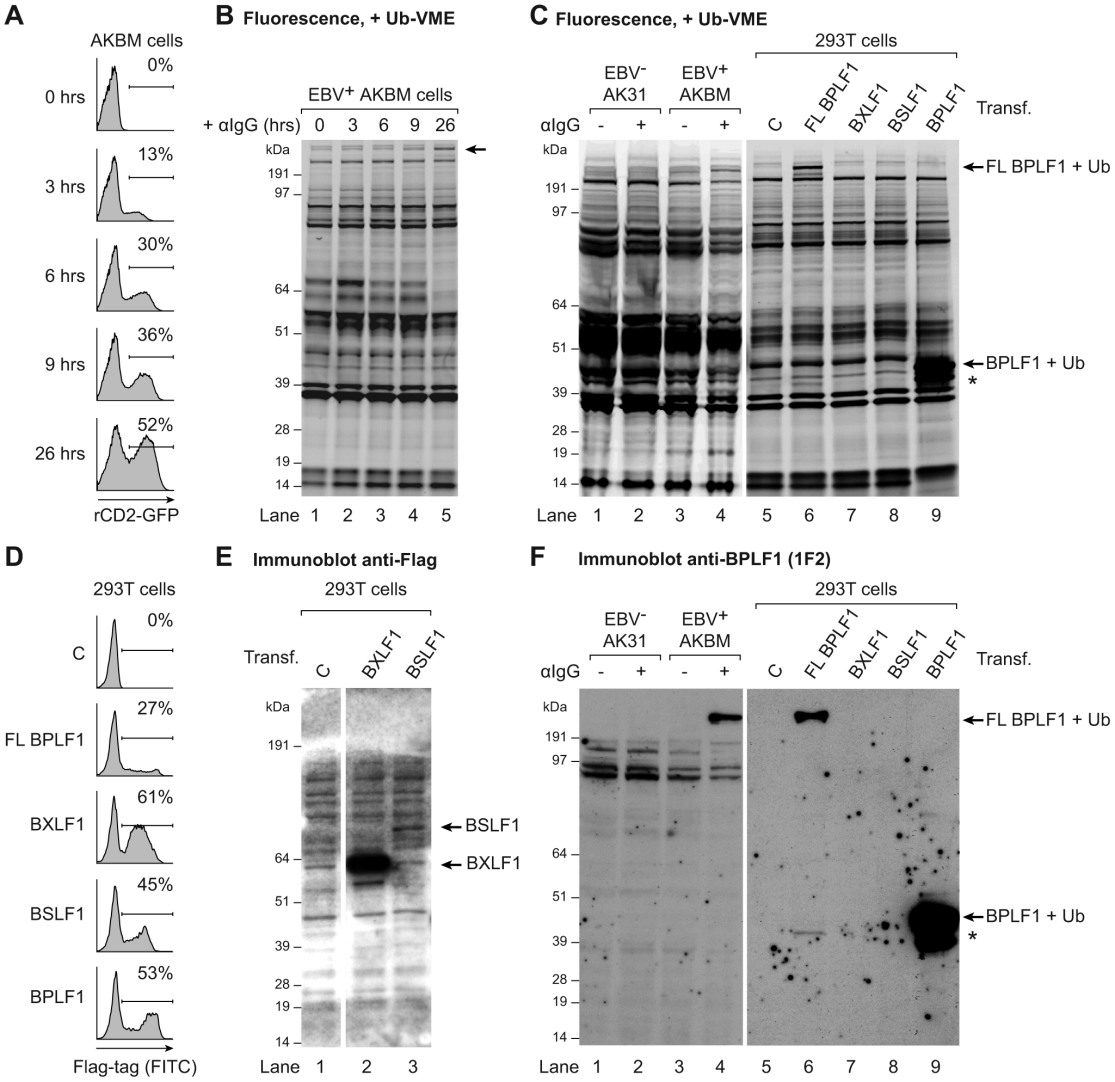
EBV BPLF1 is a DUB active during productive infection. EBV^+^ AKBM BL cells were treated with anti-human IgG (αIgG) to induce viral replication. (**a**) At the indicated times post induction, percentages of productively EBV-infected AKBM cells were determined by flow cytometric analysis of induced ratCD2-GFP reporter expression. (**b**) In post-nuclear AKBM cell lysates, active DUBs were labeled with a fluorescent Ub-VME probe, resolved by SDS-PAGE, and visualized by in-gel fluorescence imaging. The arrow indicates a band appearing in AKBM cells after 9 hours of lytic cycle induction (lanes 4 and 5). (**c**) DUB profiles of EBV^−^ AK31-rCD2-GFP cells (AK31, lanes 1 and 2), EBV^+^ AKBM cells (lanes 3 and 4), and transfected 293T cells (lanes 5–9). In parallel with the EBV^+^ AKBM cells, EBV^−^ control AK31 cells were treated with αIgG for 24 hours; this resulted in productive infection in 56% of AKBM cells; AK31 cells included a population of 45% that expressed ratCD2-GFP from a constitutive promoter irrespective of αIgG treatment (data not shown). For comparison, 293T cells were transfected with constructs encoding three (putative) EBV DUBs: BPFL1, BXLF1, BSLF1, or the 325 aa N-terminal part of BPLF1. The asterisk marks a smaller fragment observed upon transfection of 293T cells with full-length or the N-terminal part of BPLF1 (lanes 6 and 9). Left and right panels are parts of one gel displayed at different exposures. (**d**) Sixteen hours post-transfection, percentages of 293T cells expressing BPLF1, BXLF1, BSLF1 or the N-terminal fragment of BPLF1 were determined by intracellular FACS staining for the Flag-tag. (**e**) Immunoblot of part of the gel in c probed with an anti-Flag Ab to detect transfected BXLF1 and BSLF1 (sequential staining following 1F2, see in f). (**f**) Immunoblot of the gel in c probed with the BPLF1-specific mouse monoclonal Ab 1F2. Both panels are part of one gel presented at different exposures.

To determine if EBV expresses active viral DUBs, we profiled lytically and latently EBV-infected B cells. Productive infection was induced in the latent EBV^+^ B cell line AKBM by treatment with anti-human IgG antibodies (Abs) to crosslink the B cell receptor and thereby reactivate EBV [33]. At 26 hours of induction, up to 52% of cells had entered the lytic cycle, as visualized by rat CD2-GFP reporter expression (Fig. 1a). Post-nuclear cell lysates were incubated with a fluorescently labeled ubiquitin (Ub)-based, active-site directed probe (Ub-VME) [36]. This probe interacts with the active site cysteines of DUBs to form a covalent adduct. The fluorescently-labeled active DUB can then be visualized following SDS-PAGE by in-gel imaging. Using this approach, numerous Ub-VME-reactive species were visible in latently infected AKBM cells, likely representing cellular DUBs (Fig. 1b, lane 1). After 9 and 26 hours of anti-IgG treatment, an additional high molecular weight band of >200 kDa appeared, suggestive of induced expression of a DUB at late times of productive EBV infection (arrow, lanes 4 and 5, and Fig. S1 for enlarged view).

To investigate whether this DUB was virus-encoded, we compared the DUB profiles of EBV^+^ B cells (AKBM) to those of control EBV^−^ B cells (AK31). Both cell lines were either mock-treated or treated with anti-IgG for 24 hours and active DUBs were visualized in post-nuclear lysates (Fig. 1c, lanes 1–4). The high molecular weight species reacting with the Ub-VME probe in productively infected AKBM cells (Fig. 1c, lane 4) was not observed in the EBV^−^ AK31 cells, even after treatment with anti-IgG (Fig. 1c, lanes 2). This suggests that the active DUB appearing in productively infected B cells is encoded by EBV.

Earlier, three EBV proteins were reported to display DUB activity *in vitro*: BPLF1, BSLF1, and BXLF1 [31], [32]. To examine whether any of these corresponded to the active DUB observed in productively infected B cells, the DUB profile of EBV-infected B cells was compared to those of 293T cells that transiently expressed the Flag-tagged BPLF1, BSLF1, or BXLF1 protein (Fig. 1c). By intracellular FACS staining, the BXLF1 and BSLF1 proteins were detected in around 50% of cells (Fig. 1d). In contrast, full-length BPLF1 (FL BPLF1) was detectable in only up to 27% of cells, likely due to the large size of the protein (∼3150 amino acids). So far, others have used N-terminal fragments of the BPLF1 protein comprising the catalytic site for DUB activity in their studies (varying in length from 205 to 325 aa) [38]–[41]. Thus, we transfected 293T cells in parallel with a vector encoding the N-terminal 325 amino acids of BPLF1, which yielded higher transfection efficiencies (up to 53% positive cells, Fig. 1d) than full-length BPLF1.

Ub-VME-labeled bands corresponding to the BXLF1 or BSLF1 proteins were not observed by DUB profiling of transfected 293T cells (Fig. 1c, lanes 7 and 8), indicating that these display very little or no DUB activity. Both EBV proteins contain nuclear localization signals and (a proportion of) these proteins are present in the nucleus. Although intracellular FACS staining (Fig. 1d) demonstrated expression of Flag-BSLF1 as well as Flag-BXLF1 proteins in transfected cells, they could have been absent from the post-nuclear cell lysates used for DUB profiling. However, Flag-specific immunoblots of the gels used for fluorescence imaging showed that both BSLF1 and BXLF1 were present in the post-nuclear lysates (Fig. 1e, lanes 2 and 3). In our hands neither BSLF1 nor BXLF1 exerted *in vitro* cleavage of fluorogenic Ub-AMC substrates or reduction in Ub-protein adducts in 293T cells expressing HA-ubiquitin (data not shown). Furthermore, we did not find specific Ub-VME-reactive proteins corresponding to the sizes of these two proteins in productively EBV-infected AKBM cells (Fig. 1c, lane 4). These combined results imply that BSLF1 and BXLF1 do not behave as active DUBs when expressed in cells.

In contrast, transfection of full-length BPLF1 in 293T cells resulted in expression of an active DUB visible as a specific Ub-VME reactive band migrating over 200 kDa (Fig. 1c, lane 6). The size of this band corresponded to that observed in lytically infected AKBM cells (lane 4), suggesting that the large DUB expressed during productive EBV infection is BPLF1. Upon cellular expression of full-length BPLF1, an additional smaller active DUB fragment was visible around 42 kDa (asterisk, lane 6). Interestingly, transfection of the N-terminal 325 aa of BPLF1 also yielded this smaller fragment, in addition to the expected band at 46 kDa (corresponding to 36 kDa of BPLF1 covalently linked to the Ub-VME probe of 10 kDa, lane 9). The active fragment of ∼42 kDa (corresponding to ∼280 aa) could have arisen upon alternative expression or processing of BPLF1. Others have also reported the presence of a smaller fragment upon expression of an N-terminal part of BPLF1 [38], [42]; here, we show for the first time that a similar fragment is generated upon expression in 293T cells of full-length BPLF1.

To enable the study of BPLF1 protein expression during natural EBV infection (i.e. without (Flag-)epitope tag), we generated monoclonal Abs against two peptides in the N-terminal domain of this protein (based on the B95.8 EBV strain, see Fig. S2a). In immunoblots of transfected 293T cells, mAb 1F2 (epitope: BPLF1 residues 78–94) reacted with N-terminal fragments of BPLF1 of both the B95.8 and Akata EBV strains (Fig. S2b, Fig. 1f lane 9, and Fig. S3b lane 1). Importantly, this BPLF1-specific Ab visualized the full-length protein and shorter fragment in transfected 293T cells and also the active DUB expressed in productively infected AKBM cells (Fig. 1f, lanes 6 and 4, respectively, and Fig. S3), indicative of BPLF1 being expressed during the EBV lytic cycle.

Thus, for two out of the three putative EBV DUBs, BSLF1 and BXLF1, we did not find indications for DUB activity in cells. The large tegument protein BPLF1, on the other hand, appeared to interact with a functional probe both in transfected 293T cells and in EBV-producing B cells, implying that it acts as an active DUB during late times of productive EBV infection. Since both full-length BPLF1 and the N-terminal part yielded the same active smaller fragment, a construct expressing the N-terminal 325 aa of BPLF1 was used in further assays.

### BPLF1 interferes with TLR-mediated NF-κB activation

**Figure 2:**
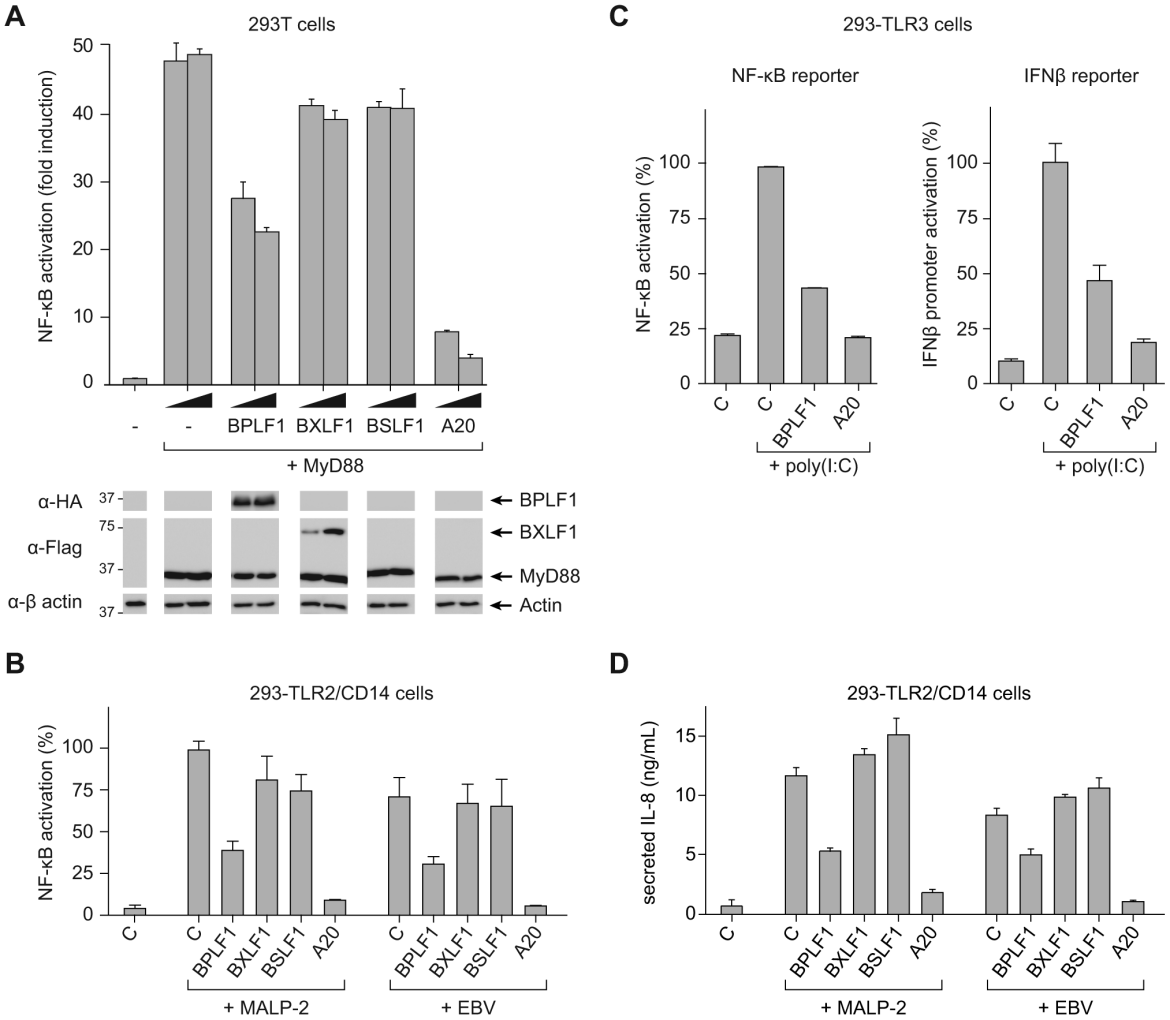
BPLF1 interferes with TLR-mediated NF-κB activation. 293T cells were transiently transfected with a firefly luciferase reporter construct responsive to either NF-κB activation or IFNβ promoter activity, an HSV TK-promoter driven Renilla luciferase plasmid (for normalization), and plasmids encoding HA-BPLF1, Flag-BXLF1, Flag-BSLF1 or cellular control DUB A20. (**a**) An NF-κB responsive firefly luciferase reporter and 4 or 8 ng of vectors encoding the EBV or control proteins were cotransfected in 293T cells and the TLR signaling cascade was initiated by expressing adaptor protein MyD88. Sixteen hours post-transfection, cells were lysed and luciferase activity was determined. In addition, protein expression was analyzed in post-nuclear cell lysates by immunoblot using anti-HA and anti-Flag Abs; β-actin served as loading control. (**b**) 293-TLR2/CD14 cells were cotransfected with an NF-κB responsive reporter and 32 ng of plasmids encoding the indicated proteins. Starting at 16 hours post-transfection, cells were stimulated with 10 ng/mL MALP-2 or 6.5×10^6^ EBV particles/mL for 7 hours, after which they were lysed and luciferase activity was measured. (**c**) Similarly, 293-TLR3 cells were cotransfected with 16 ng of the indicated genes together with an NF-κB responsive reporter (left panel) or a reporter under control of the IFNβ promoter (right panel). Starting 16 hours post-transfection, cells were stimulated for 7 hours with 10 µg/mL poly(I∶C), after which they were lysed and luciferase activity was determined. Data are presented as percentages firefly luciferase activity relative to stimulated control samples and normalized for transfection efficiency using *Renilla* luciferase values, mean ± SD. (**d**) 293-TLR2/CD14 cells were transfected with empty control vector or plasmids encoding EBV BPLF1 or cellular A20. Starting at 24 hours post-transfection, cells were stimulated with 10 µg/mL MALP2 or 6.5×10^6^ EBV particles/mL for 8 hours. IL-8 secretion in the culture supernatants was determined by ELISA.

In view of the critical role of (de)ubiquitination in regulating TLR signal transduction, we next addressed the question: does EBV exploit virus-encoded DUBs to evade innate immune activation signaled through TLRs? To this end, we monitored NF-κB activity in 293T cells transfected with an NF-κB-responsive firefly luciferase reporter. Co-transfection of MyD88, the adaptor molecule used by all TLRs except TLR3, resulted in robust activation of NF-κB (∼50-fold induction of luciferase activity compared to control cells; Fig. 2a). Upon additional introduction of each of the three EBV proteins in these cells, expression of BPLF1 resulted in a dose-dependent inhibition of NF-κB activation. BXLF1 and BSLF1 did not substantially affect NF-κB activation following MyD88 signaling (Fig. 2a), in line with their undetectable DUB activity in cells. Immunoblots revealed comparable amounts of MyD88 protein in each sample. The BPLF1 and BXLF1 proteins were clearly detectable in the post-nuclear cell lysates. BSLF1 protein levels, however, appeared much lower (data not shown), representing low expression and/or a predominantly nuclear localization of this EBV protein. Overexpression of the cellular DUB A20 in these cells, as a control known to inhibit TLR signaling, was also difficult to detect by immunoblot (data not shown), yet strongly counteracted NF-κB activation (Fig. 2a).

As a more physiological alternative to initiating TLR signaling by transfection of MyD88, we stimulated 293 cells that stably expressed TLR2 and CD14 (293-TLR2/CD14) or TLR3 (293-TLR3) with their cognate ligands. TLR2-mediated NF-κB activation upon MALP-2 stimulation was inhibited by BPLF1 and A20, but not by BXLF1 or BSLF1 (Fig. 2b). In contrast to the MyD88-dependent TLR2, TLR3 activates NF-κB through a different adaptor molecule, TRIF. This pathway, stimulated by poly(I∶C) treatment, was also repressed by EBV BPLF1 as well as by the control DUB A20 (Fig. 2c, left panel). In addition, TLR3 stimulation induces type I IFN production. Activation of an IFN β-reporter was also strongly reduced upon expression of BPLF1 – and control A20 – in poly(I∶C)-stimulated 293-TLR3 cells (Fig. 2c, right panel).

Finally, we tested what the consequences would be of EBV DUB expression for TLR activation of endogenous proinflammatory cytokine production (Fig. 2d). Stimulation of 293-TLR2/CD14 cells with MALP-2 ligand induced the production of IL-8 and this was counteracted by expression of BPLF1. As EBV has also been reported to activate TLR2 [16], we performed the same experiments using viral particles rather than MALP-2 to stimulate TLR2 signaling. In response to EBV particles, both NF-κB activation and IL-8 production occurred to levels comparable to those observed for MALP-2 stimulation in 293-TLR2/CD14 cells (Fig. 2b and d). No NF-κB activation or IL-8 production was observed upon stimulation of TLR2-negative 293-CD14 cells (not shown) indicating that the EBV-induced response was mediated by TLR2. Moreover, BPLF1 reduced NF-κB-luciferase and IL-8 production also upon TLR stimulation by EBV; BXLF1 and BSLF1 had no influence in this context (Fig. 2b and d). These results indicate that EBV BPLF1 inhibits proinflammatory cytokine production by cells in which the innate immune system has been activated by viral particles.

Taken together, we found that one of the (putative) EBV DUBs, the large tegument protein BPLF1, counteracts both MyD88- and TRIF-dependent TLR signal transduction pathways.

### The ability of BPLF1 to modulate TLR signaling correlates with its deubiquitinase activity

To assess whether BPLF1 relies on deubiquitination to interfere with TLR signaling, we used three previously reported BPLF1 mutants (C61A, D86/90R, and D86/90G). Replacing the catalytic cysteine residue 61 by an alanine in BPLF1_C61A_ has been shown to abolish enzymatic activity [31]. Besides acting as a DUB, BPLF1 is capable of hydrolyzing Nedd8 conjugates and affects the major cellular neddylated proteins, the cullins [38], [39]. Two mutants, BPLF1_D86/90R_ and BPLF1_D86/90G_, contain altered charged residues at the protein surface that almost completely prevent interaction with the cullin proteins and, therefore, no longer deneddylate them. While the catalytic site C61 residue is unaltered, enzymatic activities of these two mutants are differentially affected, probably due to conformational changes in the protein [39].

**Figure 3:**
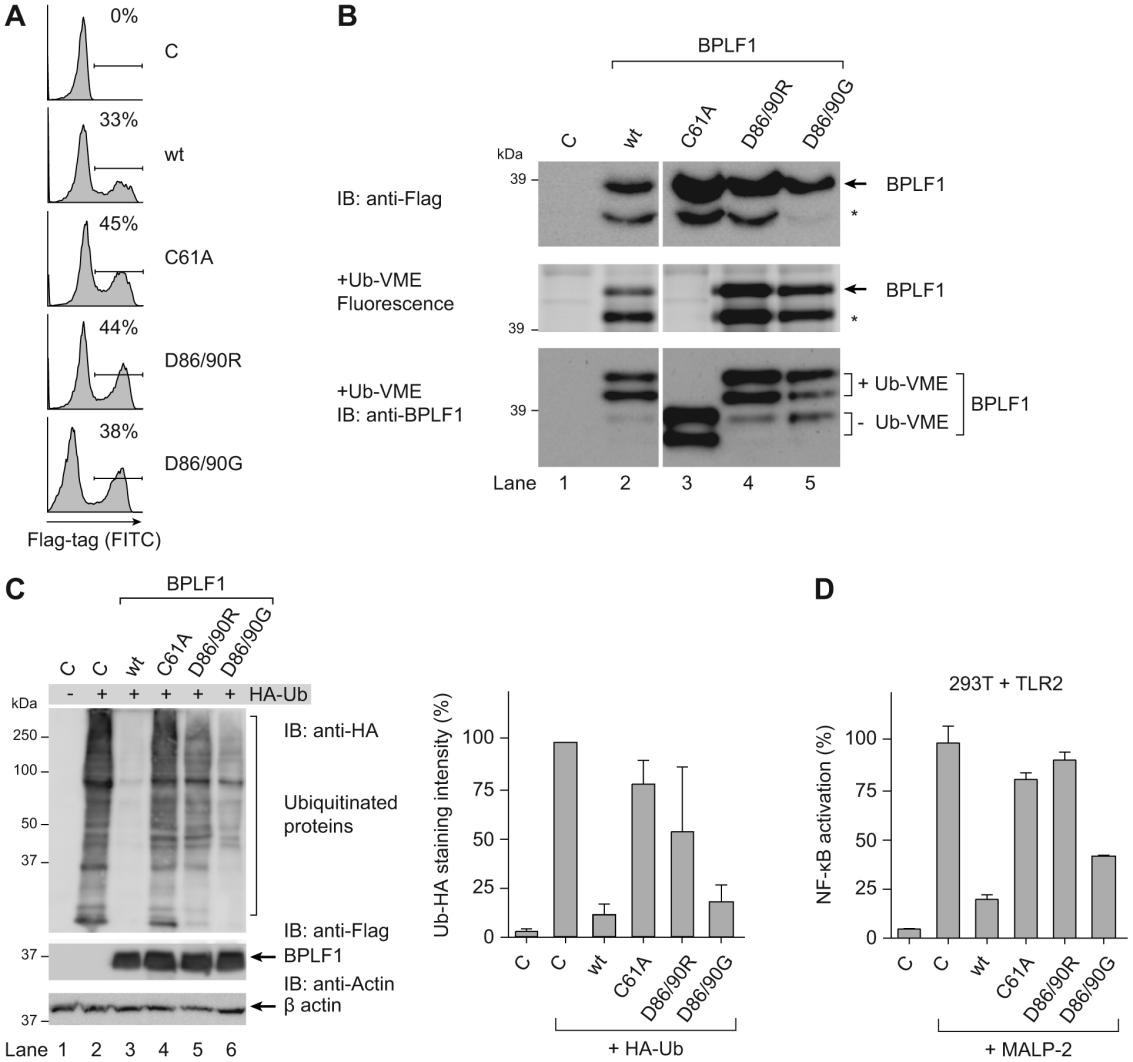
BPLF1-mediated modulation of TLR signaling correlates with deubiquitinase activity. 293T cells were transiently transfected with an empty vector control or plasmids encoding Flag-tagged BPLF1 or mutants thereof (BPLF1_C61A_, BPLF1_R86/R90_, or BPLF1_G86/G90_). (**a**) Sixteen hours post-transfection, the percentage of cells expressing BPLF1 was determined by intracellular flow cytometric analysis using an anti-Flag Ab. (**b, upper panel**) Post-nuclear cell lysates were prepared and immunoblotted using an anti-Flag Ab. The asterisk marks a smaller active fragment observed upon expression of BPLF1. (**b, middle panel**) Active DUBs in these cell lysates were labeled using the fluorescent Ub-VME probe (causing a concomitant size increase of 10 kDa) and visualized by in-gel imaging. (**b, lower panel**) Immunoblot of the gel in c probed with BPLF1-specific Ab 2E5. Unlabeled BPLF1 migrates at 36 kDa and 32 kDa, while the upper two bands of 46 kDa and 42 kDa represent probe-bound BPLF1. (**c**) 293T cells were cotransfected with HA-ubiquitin and BPLF1 variants. After 40 hours, total lysates were prepared and immunoblotted for HA-Ub-protein adducts using an anti-HA Ab (left panel; right panel shows the quantification of average staining intensities from four independent experiments). Expression levels of BPLF1 variants were assessed using an anti-Flag Ab; β-actin served as loading control. (**d**) 293T cells were cotransfected with vectors encoding TLR2, an NF-κB responsive firefly luciferase reporter, HSV TK-driven *Renilla* luciferase for normalization, and BPLF1 variants (16 ng). Following treatment with 10 ng/mL MALP-2 for 7 hours, cells were lysed and luciferase activity was measured. The data are presented as percentage firefly luciferase activity relative to stimulated control sample and normalized for transfection efficiency using *Renilla* luciferase values, mean ± SD.

Transient transfection of these BPLF1 variants in 293T cells resulted in similar protein expression levels (Fig. 3a, FACS; Fig. 3b, immunoblot). In agreement with earlier reports, DUB labeling with the fluorescent Ub-VME probe was abrogated by the C61A catalytic site mutation, whereas BPLF1_D86/90R_ and BPLF1_D86/90G_ maintained reactivity (Fig. 3b middle and lower panel, in gel-fluorescence and subsequent immunoblot, respectively). The immunoblot depicted in figure 3b (lower panel) clearly shows the larger and smaller BPLF1 fragments (lane 3) and their 10 kDa shift upon covalent attachment of the active site probe (lanes 2, 4, and 5). Differences among the BPLF1 variants became more apparent in a cellular assay for DUB activity, in which ubiquitinated proteins in 293T cells were visualized through transfection with HA-tagged ubiquitin (Fig. 3c). In this assay, potent deubiquitination was mediated by wild-type (wt) BPLF1 (lane 3) and, to a slightly lesser extent, by BPLF1_D86/90G_ (lane 6); BPLF1_D86/90R_ had largely lost its capacity to remove HA-ubiquitin from cellular proteins (lane 5), as did the catalytic mutant BPLF1_C61A_ (lane 4).

We next examined the effects of expressing wt BPLF1 or the three mutants on TLR signaling. To this end, 293T cells were transfected to transiently (co-)express the TLR2 protein (Fig. 3d). NF-κB activation following MALP-2 treatment of these cells was inhibited by wt BPLF1 and, to a lesser extent, by BPLF1_D86/90G_. In contrast, both the catalytically inactive mutant BPLF1_C61A_ as well as BPLF1_D86/90R_ did not reduce NF-κB activation. As TLR interference by these BPLF1 variants (Fig. 3d) correlates with their reactivity towards intracellular Ub-protein adducts (Fig. 3c), we conclude that the ability of BPLF1 to downmodulate TLR signaling depends on its intracellular DUB activity.

### BPLF1 acts at multiple levels in the TLR signal transduction cascade

Regulation of TLR responses involves K48- and K63-linked ubiquitination of various signaling intermediates. To delineate where EBV BPLF1 acts to inhibit TLR signaling, we performed the following experiments.

**Figure 4:**
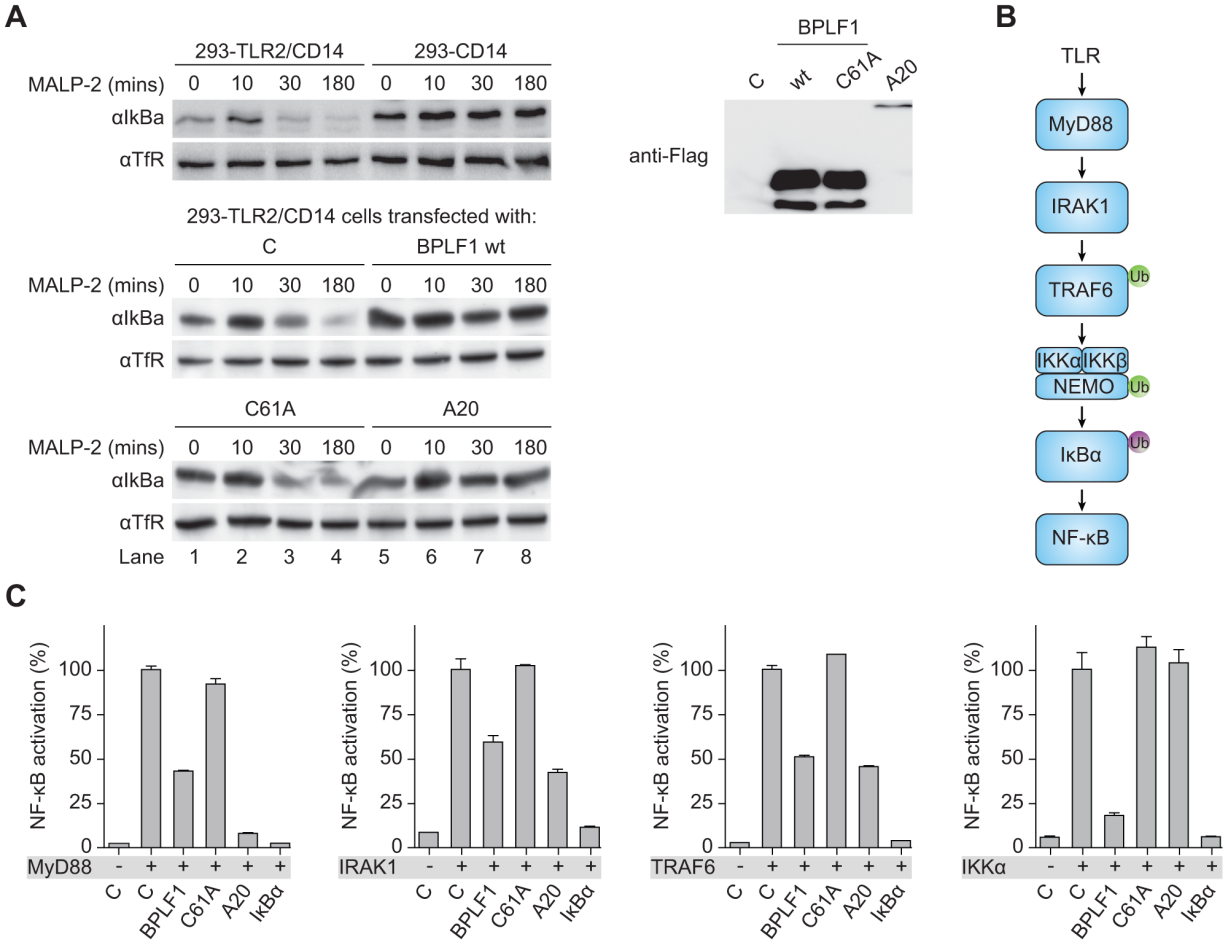
BPLF1 interferes with TLR signal transduction at multiple levels. (**a**) 293-TLR2/CD14 cells and control 293-CD14 cells were transiently transfected with plasmids encoding Flag-tagged BPLF1, catalytically inactive mutant BPLF1_C61A_, or cellular control DUB A20. At 40 hours post-transfection, cells were treated with 10 ng/mL MALP-2 for indicated time periods and IκBα levels in post-nuclear cell extracts were determined by immunoblotting with an anti-IκBα Ab. Expression of transfected Flag-tagged proteins was assessed by immunoblotting with an anti-Flag Ab; TfR served as loading control. (**b**) Schematic representation of the signaling cascade that induces NF-κB following TLR activation. Ub in green is K63-linked, Ub in purple is K48-linked. (**c**) 293T cells were cotransfected with an NF-κB responsive firefly luciferase reporter, HSV TK promoter-driven Renilla luciferase for normalization, and plasmids expressing BPLF1, BPLF_C61A_, or controls IκBα and A20 (13.5 ng). TLR signaling was induced by expression of activator proteins MyD88, IRAK1, TRAF6, or IKKα. After 16 hours of transfection, luciferase activity was measured. Results are depicted as percentage firefly luciferase activity normalized using *Renilla* luciferase values relative to stimulated control sample, mean ± SD.

Transcription of type I IFNs and proinflammatory cytokines is induced by NF-κB after its translocation into the nucleus upon release from the cytosolic inhibitor IκBα. Downstream of TLR stimulation, IκBα is targeted for proteasomal degradation by K48-linked ubiquitination. To investigate whether BPLF1 interferes with this process, we assessed IκBα levels in 293-TLR2/CD14 cells (Fig. 4a). In response to the TLR2 ligand MALP-2, IκBα degradation became apparent in TLR2-expressing cells after 30 minutes of stimulation and this was counteracted by the cellular DUB A20. Cellular expression of the EBV DUB BPLF1 likewise prohibited IκBα degradation, whereas the catalytically inactive mutant BPLF1_C61A_ did not. Thus, BPLF1 interferes with TLR-mediated NF-κB signaling at, or upstream of, the level of IκBα degradation in a manner dependent on its DUB activity.

Several signaling intermediates upstream of IκBα are regulated through K63-linked ubiquitination (Fig. 4b). To dissect these steps, we stimulated the TLR signaling pathway at different levels by transfection of 293T cells with the activator proteins MyD88, IRAK1, TRAF6, or IKKα, and monitored ensuing NF-κB activation in luciferase assays (Fig. 4c). Feasibility of this approach is illustrated by two controls. Firstly, IκBα blocks NF-κB activation downstream of IKKα, and IκBα expression therefore inhibited responses induced by MyD88 down to IKKα. Secondly, A20 is known to deubiquitinate TRAF6; A20 expression inhibited NF-κB activation mediated by MyD88-, IRAK1-, and TRAF6, but not IKKα. EBV BPLF1 reduced signaling induced by expression of all four signaling intermediates. This inhibitory effect occurred through deubiquitination, since the catalytic mutant BPLF1_C61A_ did not alter NF-κB activation (Fig. 4c). These data point to the EBV DUB targeting ubiquitinated signaling molecules at or upstream of the IKKα/IKKβ/NEMO complex.

**Figure 5:**
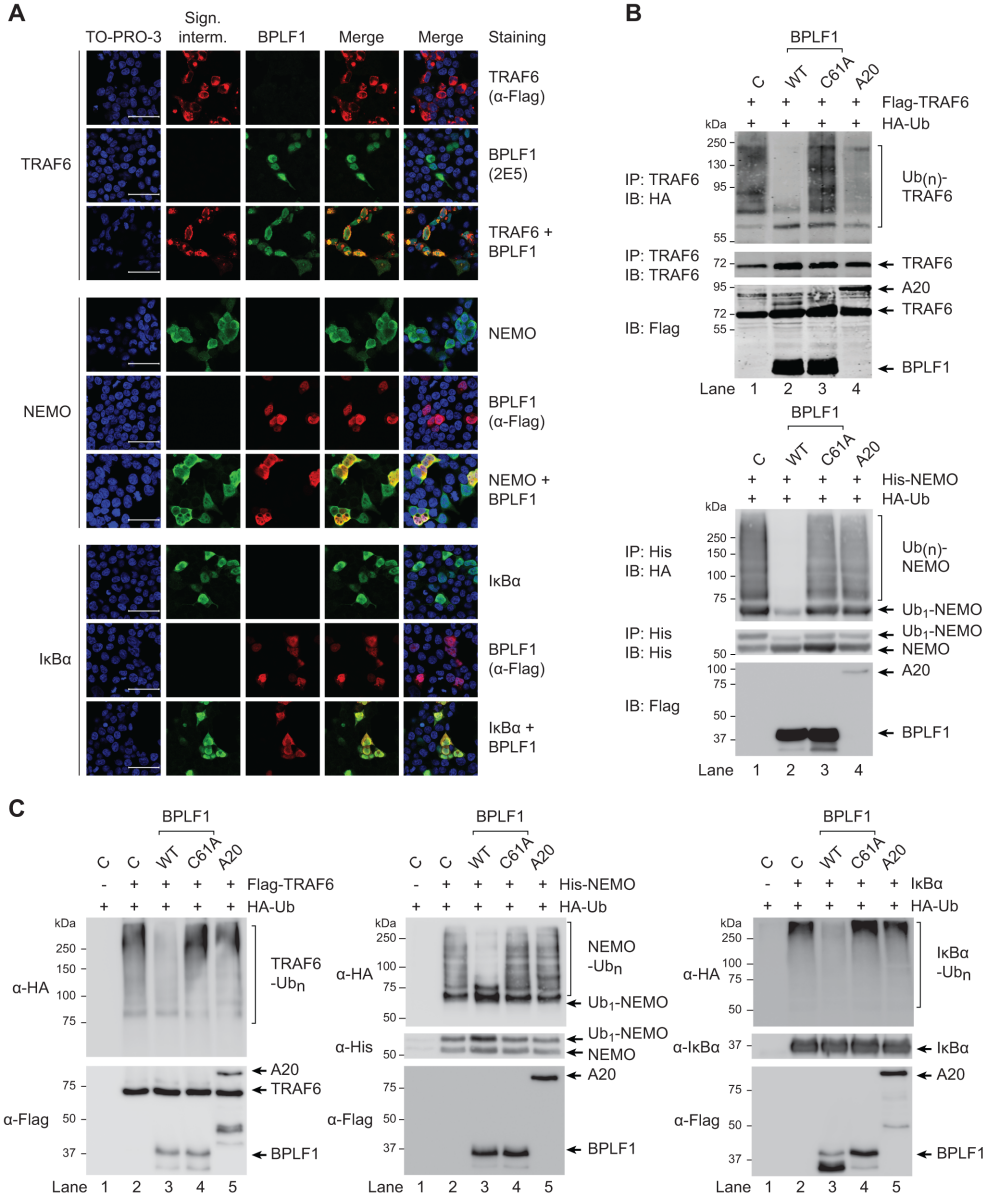
EBV DUB BPLF1 can target K63- and K48-ubiquitinated signaling intermediates. (**a**) Fluorescence micrographs of 293T cells that were cotransfected with plasmids encoding Flag-TRAF6 and HA-BPLF1 (upper panel), NEMO and Flag-BPLF1 (middle panel), or IκBα and Flag-BPLF1 (lower panel). Sixteen hours post-transfection, signaling intermediates (column 2) and BPLF1 (column 3) were labeled using anti-Flag (red; for TRAF6 in upper panel; for BPLF1 in middle and lower panels), anti-BPLF1 (green), anti-NEMO (green), and anti-IκBα (green) Abs as indicated. Nuclei were visualized using TO-PRO-3 (blue, column 1). Column 4 and 5 show merged pictures from red and green channels without and with nuclear stain (blue), respectively. Scale bar 50 µm. (**b**) 293T cells were cotransfected with plasmids encoding either Flag-TRAF6 (upper panel) or His-NEMO (lower panel) together with HA-ubiquitin and BPLF1, BPLF1_C61A_, or A20. Expression of Flag-tagged proteins was assessed by immunoblotting (IB) with an anti-Flag Ab. TRAF6 and NEMO were precipitated (IP) from post-nuclear lysates and detected using an anti-TRAF6 or anti-His Ab, respectively. HA-tagged ubiquitin adducts were visualized on immunoblot using anti-HA Abs. (**c**) 293T cells were cotransfected with plasmids encoding HA-ubiquitin together with Flag-TRAF6 (left panel), His-NEMO (middle panel), or IκBα (right panel). IκBα-transfected cells were additionally treated with TNFα and the proteasome inhibitor MG132 for 2 hours before lysis. Twenty-four hours post-transfection, TRAF6, NEMO, and IκBα were precipitated from post-nuclear cell lysates and incubated *in vitro* with separately purified Flag-BPLF1, Flag-BPLF1_C61A_, or Flag-A20 proteins for 4 hours at 37°C. Flag-tagged DUBs were detected by immunoblotting with anti-Flag Ab and signaling intermediates were visualized using anti-Flag (TRAF6), anti-His (NEMO), or anti-IκBα Abs. HA-ubiquitin adducts were detected using an anti-HA Ab.

Confocal microscopy was employed to study cellular colocalization of the EBV-encoded DUB and three ubiquitinated intermediates in the TLR signaling pathway, a prerequisite for their direct deubiquitination by BPLF1. 293T cells transfected with BPLF1 and either TRAF6, NEMO, or IκBα were fixed and permeabilized and both EBV and signaling proteins were visualized with specific antibodies. Colocalization in the cytoplasm was observed for BPLF1 with TRAF6 and with NEMO (Fig. 5a). Transfection of IκBα results in nuclear localization of this signaling intermediate [43] and, therefore, colocalization with BPLF1 was detected in the nucleus (Fig. 5a). We next determined whether cytoplasmic colocalization with BPLF1 affected K63-linked polyubiquitination of TRAF6 or NEMO, by comparing cells expressing TRAF6 or NEMO in the absence or presence of DUBs. Immunoprecipitation from post-nuclear lysates of 293T cells co-expressing HA-ubiquitin revealed a smear of polyubiquitinated TRAF6 (Fig. 5b, upper panel lane 1) and polyubiquitinated NEMO (Fig. 5b, lower panel lane 1). Co-expression of wt BPLF1 in these cells caused a marked reduction in the amounts of ubiquitinated TRAF6 and NEMO (lanes 2), which was not induced by the catalytically inactive DUB mutant BPLF1_C61A_ (lanes 3). As a positive control, A20 also reduced poly-ubiquitination of TRAF6 (upper panel lane 4), but not of NEMO (lower panel lane 4). These data strongly indicate that cytoplasmic BPLF1 can remove K63-linked ubiquitin moieties from TRAF6 and NEMO.

Finally, to directly assess if BPLF1 can enzymatically act on both K63-linkages as well as K48-linkages present on ubiquitinated TRAF6 and NEMO, versus IκBa, we compared the *in vitro* deubiquitinase activity of isolated (in)active DUBs towards these targets (Fig. 5c). In line with the results observed upon their cellular coexpression (Fig. 5b), *in vitro* incubation of ubiquitinated TRAF6 or NEMO with wt BPLF1 resulted in removal of polyubiquitin chains, which did not occur upon incubation with the inactive mutant BPLF1 C61A (Fig. 5c, left and middle panels). The cellular DUB A20 only affected ubiquitination of TRAF6, but not of NEMO. Since K48-linked (as opposed to K63-linked) polyubiquitination results in proteasomal degradation, IκBα transfected cells were additionally stimulated with TNFα and treated with a proteasome inhibitor to obtain sufficient amounts of ubiquitinated signaling protein for evaluation in an *in vitro* DUB assay. Polyubiquitinated IκBα was also targeted by BPLF1, but not by the inactive mutant or A20 (Fig. 5c right panel), showing that the EBV DUB can interfere with K48-ubiquitin-regulated proteasomal degradation of IκBα.

Taken together, our results indicate that BPLF1 inhibits NF-κB activation by targeting multiple K48- and K63-ubiquitin-regulated steps along the TLR signaling pathway.

### BPLF1 is expressed during the late phase of productive EBV infection and is incorporated into viral particles

Conflicting information has been published on the timing of expression of the EBV DUB during productive infection, with BPLF1 being classified either as a late viral gene based on promoter consensus sequences [44], or as an early gene based on the kinetics of mRNA expression [38]. Our new BPLF1-specific monoclonal Ab provides the opportunity to address this issue by determining natural BPLF1 expression kinetics at the protein level.

**Figure 6:**
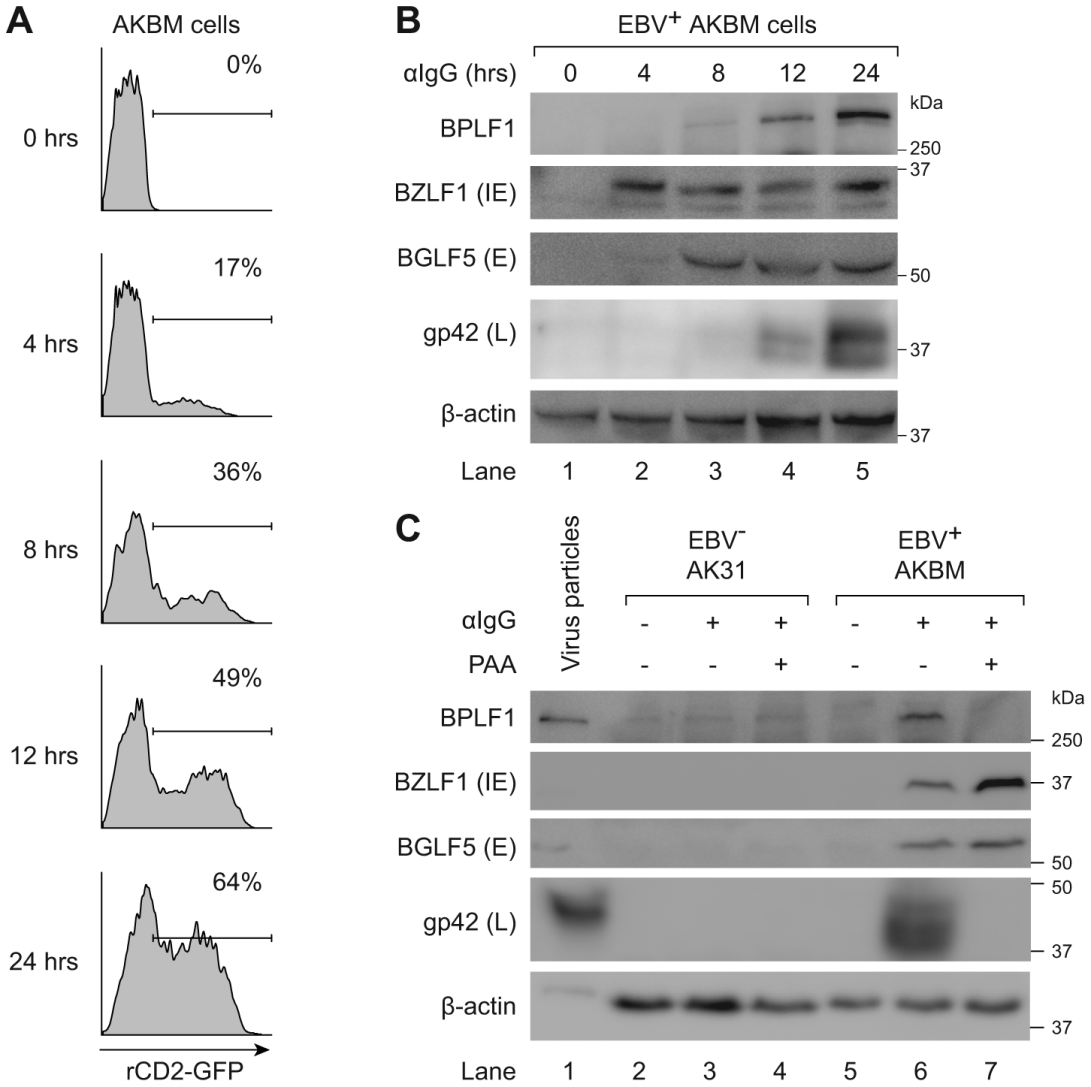
BPLF1 is expressed during the late phase of lytic EBV infection and is incorporated into viral particles. EBV^+^ AKBM cells were treated with anti-human IgG (αIgG) to induce productive infection. (**a**) At the indicated times post-induction, percentages of AKBM cells undergoing productive infection were determined by flow cytometric analysis of ratCD2-GFP reporter expression. (**b**) EBV protein expression in post-nuclear cell lysates was determined by immunoblotting with Abs specific for BZLF1 (immediate early, IE), BGLF5 (early, E), and gp42 (late, L). β-actin served as loading control. (**c**) Immunoblot of post-nuclear lysates of EBV B95.8 particles (lane 1), EBV^−^ AK31 cells (lanes 2–4), and EBV^+^ AKBM cells (lanes 5–7). EBV^+^ AKBM cells and EBV^−^ control AK31 cells were treated with αIgG Abs for 24 hours, resulting in productive infection in 26% of AKBM cells; AK31 cells expressed ratCD2-GFP constitutively in ∼40% of the population (data not shown). Phosphonoacetic acid (PAA, 300 µg/mL) treatment starting 1 hour prior to anti-IgG treatment was used to inhibit late protein expression; β-actin served as loading control.

We examined expression of EBV proteins in post-nuclear lysates of AKBM cells at various times after induction of the lytic cycle (Fig. 6a and b). The immediate early viral protein BZLF1 was readily detected at 4 hours post induction and onwards, followed by the early BGLF5 protein, and the late structural glycoprotein gp42 at 8–12 hours post induction. The BPLF1 protein appeared in productively infected AKBM cells after 8–12 hours, coincident with the timing of gp42 expression (Fig. 6b). To confirm the late kinetics of BPLF1 expression, phosphonoacetic acid (PAA) was added during anti-IgG treatment of EBV^+^ AKBM cells and the EBV^−^ AK31 control cells (Fig. 6c). PAA is an inhibitor of viral DNA replication and prevents late gene expression in productively infected B cells [45]. While BZLF1 and BGLF5 proteins were expressed in lytic AKBM cells irrespective of PAA treatment (lanes 6 and 7), the inhibitor blocked synthesis of both BPLF1 and the control late EBV protein gp42 (lane 7). Finally, we analyzed purified EBV particles (strain B95.8): in addition to the envelope protein gp42, we also found the full-length BPLF1 protein to be present (Fig. 6c, lane 1).

Taken together, these results show that the EBV DUB BPLF1 is expressed during the late phase of productive infection and is incorporated into viral particles, providing various opportunities for interference with TLR signaling.

## Discussion

In this study, we have shown that the large tegument protein BPLF1 acts as an active DUB during productive EBV infection. By removal of K63- and/or K48-linked ubiquitin chains from signaling intermediates, such as TRAF6, NEMO, and/or IκBα, BPLF1 inhibits TLR signaling through both MyD88- and TRIF-dependent pathways. This leads to reduced NF-κB activation and proinflammatory cytokine production in response to EBV.

The first herpesvirus-encoded DUB was identified by covalent binding of a ubiquitin-based active site-directed probe to the large tegument protein expressed in HSV1-infected cells [30]. The catalytic site of this DUB appeared to be conserved in the large tegument protein of all herpesviruses [31]. Based on the structure of the MCMV homologue, M48, the herpesvirus DUBs form a unique class of cysteine proteases that do not share homology with cellular deubiquitinating enzymes, except for the catalytic site triad [30], [46]. Both *in vitro* and *in vivo* studies support important roles for the conserved tegument DUBs during the viral life cycle. For example, an HCMV UL48 mutant virus yielded reduced viral titers in cultured cells [47]. A mutant of HSV, from which the homologous tegument gene UL36 had been completely deleted, did not assemble virions (late in infection) and failed to enter the nucleus (at early stages of infection) [48]. MHV-68 expressing a catalytically inactive DUB encoded by ORF64 had reduced *in vitro* replication capacity and was cleared more efficiently *in vivo* [49].

For EBV, knockout or knockdown of BPLF1 expression in lytically infected 293T or B cells, respectively, resulted in reduced virus production [38], [40], [50]. So far, one viral substrate of BPLF1 has been found: deubiquitination of the subunit BaRF1 reduced activity of the EBV ribonucleotide reductase complex [40]. Among the reported cellular targets of BPLF1 is PCNA [41], deubiquitination of which prevents recruitment of DNA damage response elements. The cullins form cellular substrates for deneddylation by BPLF1. As a consequence of active BPLF1 expression, the cullin-RING ligase neddylation cycle is interrupted leading to an S-phase cell cycle arrest that favors EBV replication [38], [39].

Although the N-terminal fragment of the homologous large tegument protein of EBV has been reported to exert DUB activity [31], [38], [40], [51], earlier attempts were unsuccessful in finding the active BPLF1 protein in EBV-infected B cells [52]. Combining our EBV lytic cycle system with a sensitive fluorescently labeled Ub-VME probe and new BPLF1-specific monoclonal Abs has now revealed an active DUB protein corresponding to full-length BPLF1 in EBV-producing B cells (Fig. 1). This complements the finding by Whitehurst *et al.* [41] of endogenous BPLF1 expression in lytically induced 293T-BAC EBV cells. While our manuscript was under revision, Gastaldello *et al.* [42] reported detection of BPLF1 as an active deneddylase as well as DUB in a related cell line, Akata-BX1, thereby confirming the reproducibility of our findings. BPLF1 is expressed as a late lytic cycle gene product and is incorporated into viral particles (Fig. 6). BPLF1's presence in virions is also supported by detection of this high molecular weight protein (indicated as large tegument protein, LTP) among EBVs structural proteins [53]. Two other reported putative EBV DUBs, BXLF1 and BSLF1, were not detectable as active DUBs in EBV-producing B cells or in transfected cells (Fig. 1). Cellular expression of full-length as well as a 325 aa N-terminal domain of BPLF1 yielded an additional smaller fragment, as also observed by others [38], [42]. Based on its size this fragment corresponds to residues 1- ∼280 of BPLF1, including the active site cysteine, and was shown to exert DUB activity (Figs. 1, 3, 5). Our preliminary data indicate that full-length BPLF1 disappeared in time (at ∼48 hours in lytic AKBM cells, Fig. S3c; from ∼72 hours in 293T cell transfected with FL BPLF1, most clearly visible as loss from the cytosol of the C-terminal HA-tag, Fig. S3b) and a smaller fragment appeared (reactive with the DUB probe and Abs specific for epitopes spanning BPLF1 aa 2–17 (peptide A) and 78–94 (peptide B); Figs. S2 and S3). This likely occurs due to processing of the full-length protein, as it coincided with appearance of N-terminal fragments of BPLF1 migrating around 32 and 25 kDa (detectable with anti-BPLF1 peptide A Ab, Fig. S3b; and with anti-Flag Ab, not shown). Shorter fragments of BPLF1 of ≤32 kDa would lack nuclear localization sequences (aa 412–418) and might localize preferably to the cytoplasm. The ∼32 kDa fragment was indeed observed in cytoplasmic (and nuclear) fractions, yet the ∼25 kDa form was restricted to the nucleus (Fig. S3b). This is in accordance with a very recent publication, where a nuclear ∼25 kDa fragment of BPLF1 was found to result from cleavage of the viral protein by caspase 1; the resulting small molecular size (≤40 kDa) could allow diffusion through the nuclear pore, which would thus facilitate deNeddylase activity towards the Cullin substrates in the nucleus [42]. We here propose that the ∼32 kDa fragment could exert DUB activity towards substrates that reside in the cytoplasm. Intracellular processing could thus regulate BPLF1's (subcellular) localization and activity. This is reminiscent of the dual role of UL36 in HSV1 infection: at late times, full-length UL36 functions as a structural/tegument protein in virion assembly from virus-producing cells, whereas at early times the protein is cleaved upon entry into newly infected cells in order to allow nuclear release of the viral DNA [54]–[56]. Since full-length and/or truncated large tegument proteins with DUB activity have additionally been reported for HCMV [57] and MHV68 [29], this may represent a general feature among these conserved viral DUBs.

In the current study, we have found that BPLF1 interferes with innate immune activation by targeting multiple intermediates along the TLR signal transduction pathway, including TRAF6, NEMO, and IκBα (Figs. 2, 4, and 5). Interestingly, Saito *et al.* very recently also reported TRAF6 to be deubiquitinated by BPLF1 [50], albeit in a different context. They reported that BPLF1 contributed to virus production by repressing NF-κB signaling, which is induced in latently infected EBV^+^ cells by the EBV latent membrane protein 1 (LMP1) and signaled through TRAF6 [50]. Although in our system, BPLF1 is not detectable in latently EBV-infected B cells, simultaneous expression of BPLF1 and LMP1 could occur in B cells during productive infection. In agreement with published results [58], low levels of LMP1 were expressed from 8 hours of lytic induction of AKBM B cells and higher levels of the shorter, lytic form of LMP1 (lyLMP1) were detected in cell lysates of 24 and 48 hours post-induction (Fig. S4). LyLMP1 does not activate NF-κB signaling itself and in fact appears to counteract (latent FL) LMP1 signaling [59], [60]. Whereas during latency LMP1 provides proliferative and survival signals through NF-κB, lyLMP1 could cooperate with BPLF1 to reduce NF-κB activation in B cells producing new virions. In our study, we addressed another implication of BPLF1's DUB function for virus-host interactions. BPLF1 inhibited NF-κB activation and proinflammatory cytokine production in response to TLR stimulation, e.g. by EBV particles (Fig. 2). In addition, we have identified likely targets downstream of TRAF6 that are relevant to this TLR inhibition: BPLF1 colocalizes with and can deubiquitinate K63-Ub-TRAF6, K63-Ub-NEMO, and K48-Ub-IκBα (Figs. 4 and 5). The mechanistic experiments were mostly performed in 293T cells with BPLF1 expressed in isolation, yet the expression levels of the signaling components we studied appeared comparable in the B cells used for productive EBV infection (data not shown). Employing mutants of BPLF1 (D86/90) that retained an unaltered catalytic site, but no longer interact with the cullins [39], we observed that inhibition of NF-κB activation correlated with DUB activity of the mutants (Fig. 3). From this, we deduced that the TLR evasive properties of BPLF1 rely on its deubiquitinase, rather than its deneddylase, activity. This places EBV BPLF1 in the arena of viral proteins that target protein ubiquitination.

As ubiquitination represents a post-translational modification that plays a regulatory role in NF-κB- and IRF-mediated immunity through protein degradation and signal transduction, it comes as no surprise that viruses have found ways to interfere with the process. On the one hand, ubiquitin conjugation can be altered in virus-infected cells. Examples include poxvirus-induced inhibition of ubiquitin-dependent degradation of IκBα via sequestration of the cellular E3 ligase involved [61] and KSHV Rta-encoded E3-ligase activity that invokes degradation of IRF7 [62]. On the other hand, increased deubiquitination imposed by viruses can also shift the balance. For instance, the cellular DUB UCHL1 appears to be upregulated by HPV16 infection leading to suppressed innate signaling through TRAF3 and NEMO [63]. The HSV tegument protein ICP0 [64] recruits the cellular DUB USP7 into the cytosol to switch off TLR signaling to NF-κB. More recently, virus-encoded DUBs were found to dampen innate responses towards both RNA viruses [65], [66] and DNA viruses, adenovirus [67] and KSHV [68]. Here we demonstrate that EBV BPLF1 also exploits its DUB activity to modulate TLR signaling.

**Figure 7:**
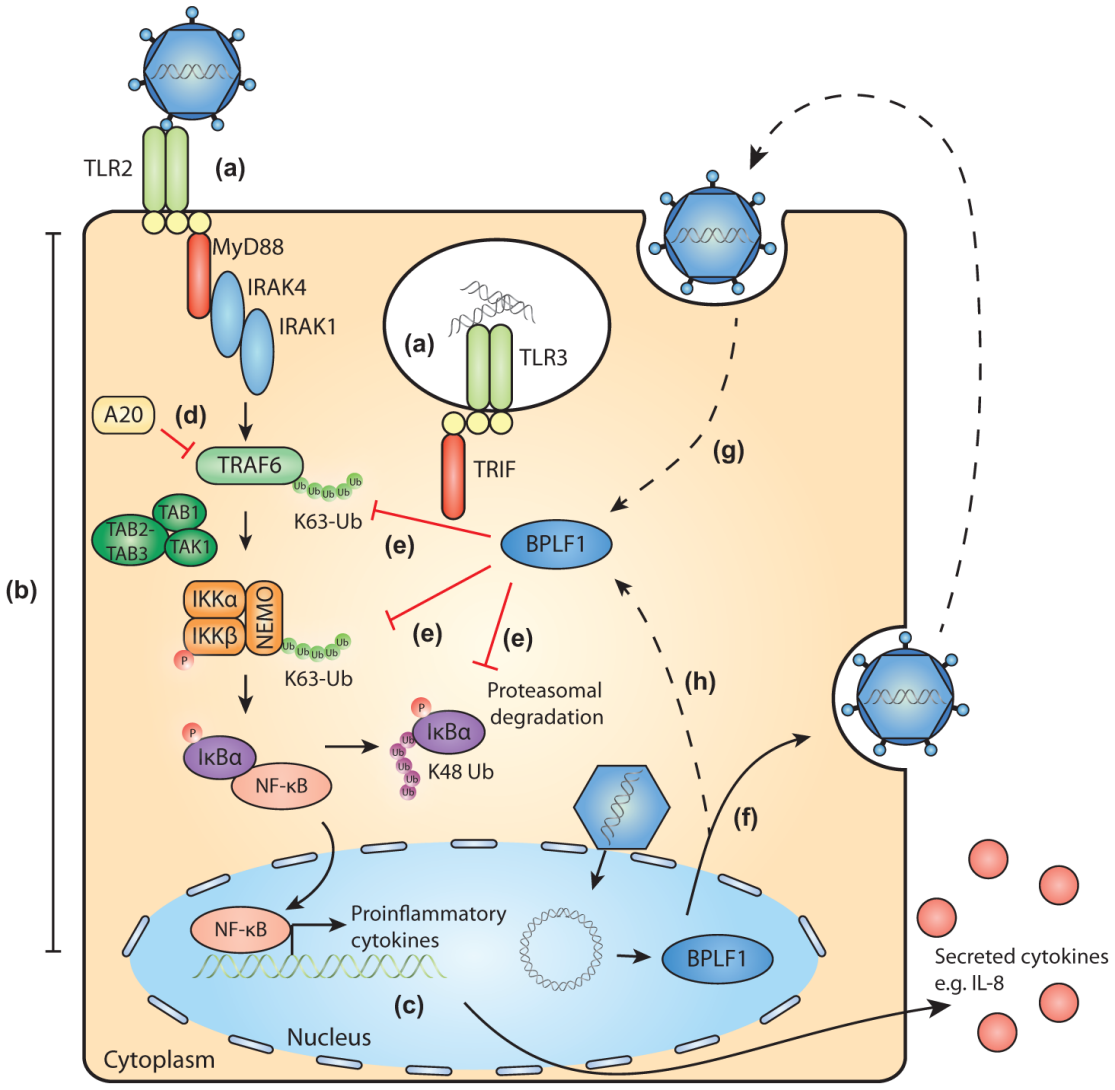
Schematic model of BPLF1-mediated TLR evasion during EBV infection. During EBV infection, EBV components are sensed by host TLRs (a). These receptors activate signaling pathways through adaptor molecules MyD88 and TRIF, culminating in activation of transcription factor NF-κB (b). NF-κB induces production of proinflammatory cytokines (c) and obstructs viral replication. TLR signaling pathways are extensively regulated by ubiquitination, a process governed by cellular ubiquitin ligases and deubiquitinases (e.g. A20, d). The EBV-encoded DUB BPLF1 counteracts TLR-mediated NF-κB activation and can interfere with K63- and K48-linked ubiquitination of signaling intermediates, for example on TRAF6, NEMO, and IκBα (e). BPLF1 is expressed as a full-length protein during the late phase of productive EBV infection, is incorporated into the tegument of viral particles (f), and can subsequently be released into newly infected cells (g). It is as yet unclear where BPLF1 is processed to yield a shorter active fragment of ca. 280 aa (see Fig. 1). Thus, (processed) BPLF1 could exert its immunomodulatory functions towards TLR signaling not only in EBV-producing B cells (h), but also during primary infection (g).

Herpesviruses are ancient viruses and have evolved in close association with their hosts, leading to acquisition of extensive strategies to perturb host immunity. A common strategy emerged from studies on viral evasion of host adaptive immune mechanisms. This is exemplified by EBV, where multiple viral proteins target the HLA class I-restricted antigen presentation pathway to ensure the optimal timing and extent of inhibition of host immunity [69]. Viruses have adopted a similar strategy to achieve innate immune evasion. Poxviruses have already been found to encode a number of inhibitors for the NF-κB pathway [70]. During productive EBV infection, the past years have also witnessed identification of a number of TLR evasion strategies. The viral tegument protein LF2 prevents activation of IRF7 [71] and the immediate-early transactivator BZLF1 negatively regulates IRF7-induced responses [72]. Of the early EBV early proteins, the viral kinase BGLF4 inhibits IRF3 activation [73] and the shutoff protein BGLF5 reduces TLR expression levels [23]. Here, we have reported on the large tegument protein BPLF1, expressed at late times of infection, downregulating TLR signaling through its DUB activity. Since tegument proteins such as BPLF1 are packaged into viral particles, they are transferred directly into newly infected cells (Fig. 7). Its close association with the EBV capsid would suggest that BPLF1 is present when PAMPs are activating innate receptors and cleavage of the N-terminal domain of BPLF1 might then allow deubiquitination to take place. It is tempting to speculate that deubiquitination by this EBV protein may exert its immunomodulatory functions in particular during primary infection. If and how this occurs awaits future elucidation.

## Supporting Information

Figure S1 **Enlarged view of the upper part of the gel depicted in** **Fig. 1b** **.** For details, see legend Fig. 1b.
(TIF)

Figure S2 **BPLF1-specific monoclonal antibodies.** Rat monoclonal Abs were generated against peptides encompassing residues 2–17 (peptide A) and residues 78–94 (peptide B) of EBV strain B95.8-encoded BPLF1. (**a**) Sequence alignment of the N-terminal parts of BPLF1 (aa 1–325) derived from the EBV strains B95.8 and Akata. An asterisk indicates the amino acid difference between these strains at position 12 (T12P). (**b**) Reactivity of two BPLF1-specific (IgG2a) mAbs 2E5 and 1F2 was tested in immunoblot. 293T cells were transiently transfected with plasmids encoding the N-terminal domains of BPLF1 from EBV strains B95.8 and Akata; transfection efficiencies were comparable (∼60% positive cells). Sixteen hours after transfection, post-nuclear cell lysates were prepared, separated by SDS-PAGE, and immunoblots were stained with an anti-Flag Ab or anti-BPLF1 Abs directed against peptide A (2E5) or peptide B (1F2). Equal loading was demonstrated by comparable band intensity observed upon staining for the Flag-tag (lanes 5 and 6); this was further supported by the staining with Ab 1F2 that reacts with an epitope identical in both EBV strains (lanes 3 and 4). The T12P amino acid difference strongly reduced 2E5-mediated detection of Akata-derived BPLF1 compared to B95.8-derived BPLF1 (lanes 1 and 2). An asterisk indicates the smaller fragment arising upon cellular expression of BPLF1. Since this 32 kDa band is recognized by both the anti-Flag Ab (tag at N-terminus) as well as the two BPLF1-reactive Abs (aa 2–17 and 78–94), it likely represents a truncated N-terminus of BPLF1 (residues 1- ∼280). (**c**) BPLF1-specific monoclonal Abs were tested in immunoprecipitation experiments. 293T cells were transfected with Flag-tagged N-terminal domains of B95.8- and Akata derived BPLF1, or Flag-BXLF1 as control. Immunoprecipitations were performed in post-nuclear lysates using monoclonal anti-BPLF1 Abs 21E9 (peptide B, lanes 2 and 4), 2E5 (peptide A, lane 3), and 1F2 (peptide A, lane 5), or anti-Flag Ab (lane 1) under native conditions or after denaturing proteins by incubating with 1% SDS as indicated. Ab 21E9 appeared ineffective in precipitating BPLF1, while 2E5 and, to a lesser extent, 1F2 precipitated B95.8 as well as Akata-derived BPLF1 under native and denaturing conditions, respectively.
(TIF)

Figure S3 **BPLF1 expression in time.** (**a**) Schematic representation of the EBV BPLF1 protein. Green boxes indicate peptides used to generate BPLF1-specific rat monoclonal Abs (2E5, anti-peptide A, aa 2–17; 1F2, anti-peptide B, aa 78–94). Asterisks denote aa substitutions of the mutants used in this study. NLS: nuclear localization signal. Numbers refer to aa positions in EBV strain B95.8. (**b**) 293T cells were transfected with plasmids encoding Flag-tagged BPLF1, HA-tagged BPLF1 or full length BPLF1 containing an N-terminal Flag-tag and C-terminal HA-tag (Flag-FL BPLF1-HA). At 24, 48, and 72 hours post-transfection, cytosolic (C) and nuclear (N) fractions were prepared and analyzed by immunoblotting with anti-BPLF1 (2E5) and anti-HA Abs. Adequate separation of cytosolic and nuclear fractions was evaluated by immunoblot analysis of cytosolic p97 and nuclear histon H3 using specific Abs. Left and right panels are part of the same gels presented at different exposures. The asterisk indicates the ∼32 kDa cytosolic fragment observed over time upon expression of FL BPLF1. (**c**) Immunoblot using BPLF1-specific Ab 1F2 shows BPLF1 expression and processing in EBV^+^ AKBM cells (lanes 4–7) and transfected 293T cells (lanes 1–3). AKBM cells were treated with anti-IgG Abs to induce productive infection and post-nuclear lysates were prepared after the indicated time periods. 293T cells were transfected with constructs encoding the N-terminal domain of BPLF1 (aa 1–325), full-length BPLF1, or an empty control plasmid (C). The asterisk indicates the smaller BPLF1 fragment observed at 24 and 48 hours after induction of productive EBV infection in AKBM cells (lanes 6 and 7) and upon expression of full-length BPLF1 in transfected 293T cells (lane 3).
(TIF)

Figure S4 **LMP1 expression during productive EBV infection in AKBM cells.** EBV^+^ AKBM cells were treated with anti-human IgG (áIgG) to induce productive infection. At the indicated times post-induction, expression of LMP1 was determined in post-nuclear cell lysates by immunoblotting using a specific Ab (lanes 2–6). LMP1 expression was observed starting 8 hours after induction of lytic infection. Lytic LMP1 (lyLMP1), an inhibitory variant of LMP1 that counteracts LMP-1 mediated activation of signaling pathways, is expressed at 24 hours post infection. Samples were the same as used for Figure 6b. EBV+ latency III cell line Jijoye constitutively expressing LMP1 was included as a positive control (lane 1).
(TIF)
