# Supplementary figures and images for: Epstein-Barr Virus Large Tegument Protein BPLF1 Contributes to Innate Immune Evasion through Interference with Toll-Like Receptor Signaling
Source: PLoS Pathog. 2014 Feb 20;10(2):e1003960. doi: 10.1371/journal.ppat.1003960 (PMC3930590; doi:10.1371/journal.ppat.1003960)

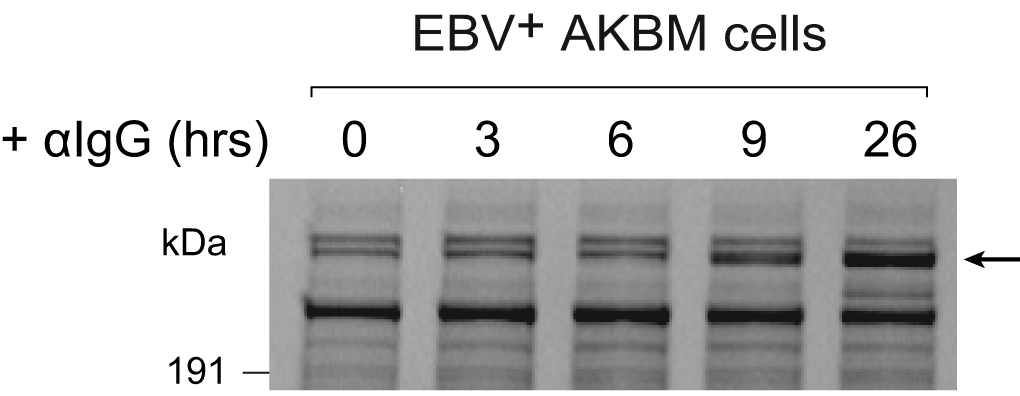

Supplement: Figure S1 — Enlarged view of the upper part of the gel depicted in Fig. 1b . For details, see legend Fig. 1b. (TIF) [file ppat.1003960.s001.tif]

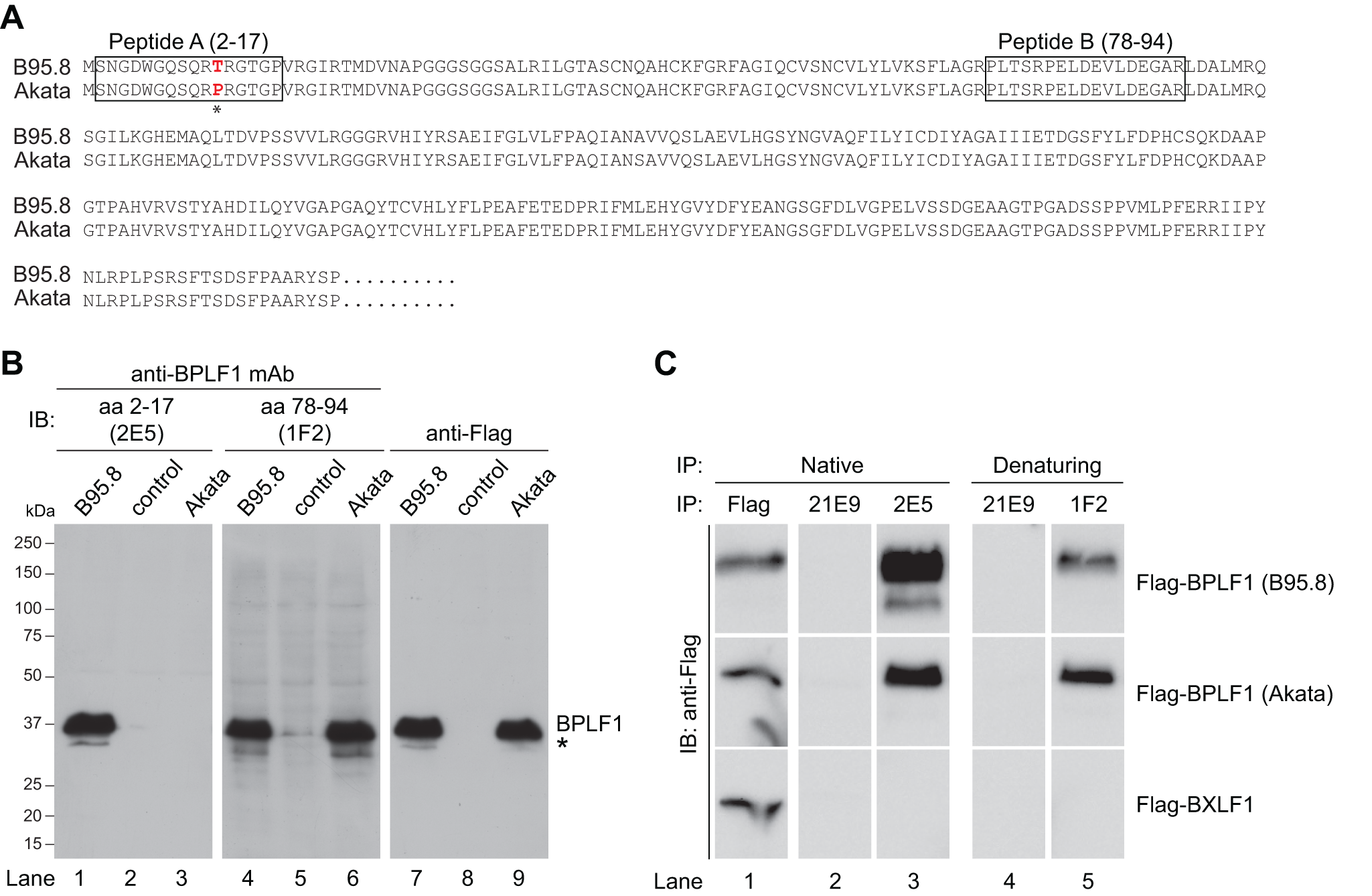

Supplement: Figure S2 — BPLF1-specific monoclonal antibodies. Rat monoclonal Abs were generated against peptides encompassing residues 2–17 (peptide A) and residues 78–94 (peptide B) of EBV strain B95.8-encoded BPLF1. (a) Sequence alignment of the N-terminal parts of BPLF1 (aa 1–325) derived from the EBV strains B95.8 and Akata. An asterisk indicates the amino acid difference between these strains at position 12 (T12P). (b) Reactivity of two BPLF1-specific (IgG2a) mAbs 2E5 and 1F2 was tested in immunoblot. 293T cells were transiently transfected with plasmids encoding the N-terminal domains of BPLF1 from EBV strains B95.8 and Akata; transfection efficiencies were comparable (∼60% positive cells). Sixteen hours after transfection, post-nuclear cell lysates were prepared, separated by SDS-PAGE, and immunoblots were stained with an anti-Flag Ab or anti-BPLF1 Abs directed against peptide A (2E5) or peptide B (1F2). Equal loading was demonstrated by comparable band intensity observed upon staining for the Flag-tag (lanes 5 and 6); this was further supported by the staining with Ab 1F2 that reacts with an epitope identical in both EBV strains (lanes 3 and 4). The T12P amino acid difference strongly reduced 2E5-mediated detection of Akata-derived BPLF1 compared to B95.8-derived BPLF1 (lanes 1 and 2). An asterisk indicates the smaller fragment arising upon cellular expression of BPLF1. Since this 32 kDa band is recognized by both the anti-Flag Ab (tag at N-terminus) as well as the two BPLF1-reactive Abs (aa 2–17 and 78–94), it likely represents a truncated N-terminus of BPLF1 (residues 1- ∼280). (c) BPLF1-specific monoclonal Abs were tested in immunoprecipitation experiments. 293T cells were transfected with Flag-tagged N-terminal domains of B95.8- and Akata derived BPLF1, or Flag-BXLF1 as control. Immunoprecipitations were performed in post-nuclear lysates using monoclonal anti-BPLF1 Abs 21E9 (peptide B, lanes 2 and 4), 2E5 (peptide A, lane 3), and 1F2 (peptide A, lane 5), or anti-Flag Ab (lane 1 [file ppat.1003960.s002.tif]

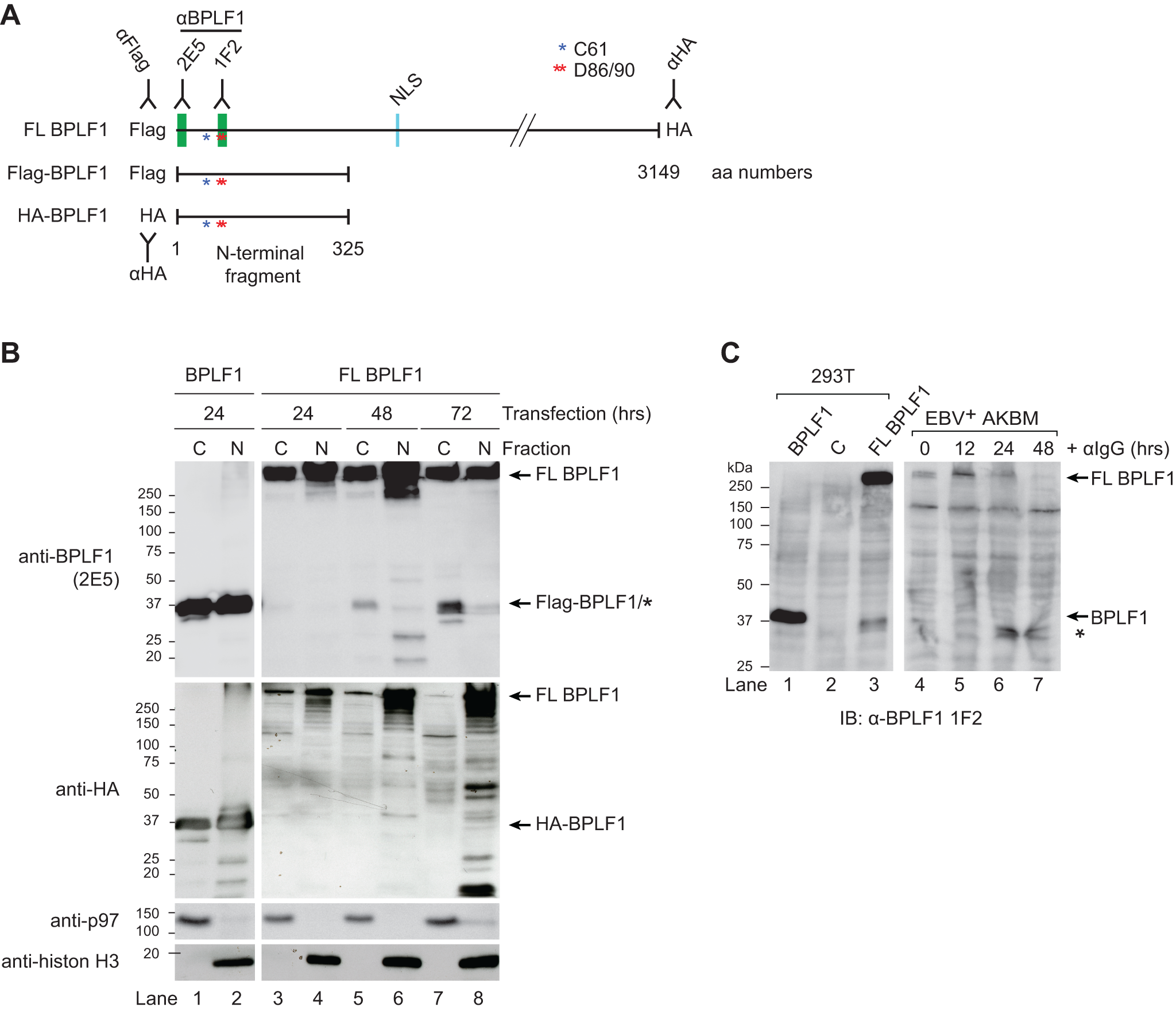

Supplement: Figure S3 — BPLF1 expression in time. (a) Schematic representation of the EBV BPLF1 protein. Green boxes indicate peptides used to generate BPLF1-specific rat monoclonal Abs (2E5, anti-peptide A, aa 2–17; 1F2, anti-peptide B, aa 78–94). Asterisks denote aa substitutions of the mutants used in this study. NLS: nuclear localization signal. Numbers refer to aa positions in EBV strain B95.8. (b) 293T cells were transfected with plasmids encoding Flag-tagged BPLF1, HA-tagged BPLF1 or full length BPLF1 containing an N-terminal Flag-tag and C-terminal HA-tag (Flag-FL BPLF1-HA). At 24, 48, and 72 hours post-transfection, cytosolic (C) and nuclear (N) fractions were prepared and analyzed by immunoblotting with anti-BPLF1 (2E5) and anti-HA Abs. Adequate separation of cytosolic and nuclear fractions was evaluated by immunoblot analysis of cytosolic p97 and nuclear histon H3 using specific Abs. Left and right panels are part of the same gels presented at different exposures. The asterisk indicates the ∼32 kDa cytosolic fragment observed over time upon expression of FL BPLF1. (c) Immunoblot using BPLF1-specific Ab 1F2 shows BPLF1 expression and processing in EBV+ AKBM cells (lanes 4–7) and transfected 293T cells (lanes 1–3). AKBM cells were treated with anti-IgG Abs to induce productive infection and post-nuclear lysates were prepared after the indicated time periods. 293T cells were transfected with constructs encoding the N-terminal domain of BPLF1 (aa 1–325), full-length BPLF1, or an empty control plasmid (C). The asterisk indicates the smaller BPLF1 fragment observed at 24 and 48 hours after induction of productive EBV infection in AKBM cells (lanes 6 and 7) and upon expression of full-length BPLF1 in transfected 293T cells (lane 3). (TIF) [file ppat.1003960.s003.tif]

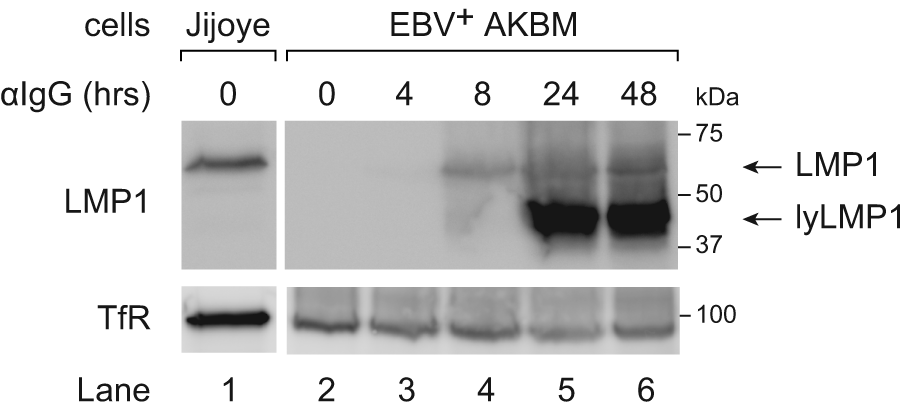

Supplement: Figure S4 — LMP1 expression during productive EBV infection in AKBM cells. EBV+ AKBM cells were treated with anti-human IgG (áIgG) to induce productive infection. At the indicated times post-induction, expression of LMP1 was determined in post-nuclear cell lysates by immunoblotting using a specific Ab (lanes 2–6). LMP1 expression was observed starting 8 hours after induction of lytic infection. Lytic LMP1 (lyLMP1), an inhibitory variant of LMP1 that counteracts LMP-1 mediated activation of signaling pathways, is expressed at 24 hours post infection. Samples were the same as used for Figure 6b. EBV+ latency III cell line Jijoye constitutively expressing LMP1 was included as a positive control (lane 1). (TIF) [file ppat.1003960.s004.tif]
